# EpCAM-targeted betulinic acid analogue nanotherapy improves therapeutic efficacy and induces anti-tumorigenic immune response in colorectal cancer tumor microenvironment

**DOI:** 10.1186/s12929-024-01069-8

**Published:** 2024-08-20

**Authors:** Debasmita Dutta, Ashique Al Hoque, Brahamacharry Paul, Jun Hyoung Park, Chinmay Chowdhury, Mohiuddin Quadir, Soumyabrata Banerjee, Arghadip Choudhury, Soumik Laha, Nayim Sepay, Priyanka Boro, Benny Abraham Kaipparettu, Biswajit Mukherjee

**Affiliations:** 1 Department of Molecular and Human Genetics, Baylor College of Medicine, Houston, TX USA; 2 Department of Coatings and Polymeric Materials, North Dakota State University, Fargo, ND USA; 3 Department of Pharmaceutical Technology, Jadavpur University, Kolkata, India; 4 CSIR- Indian Institute of Chemical Biology, Kolkata, India; 5 Department of Psychology and Neuroscience Program, Central Michigan University, Mount Pleasant, MI 48859 USA; 6 Department of Chemistry, Jadavpur University, Kolkata, India; 7 Present Address: Department of Medical Oncology, Dana Farber Cancer Institute, Boston, MA USA; 8 Harvard Medical School, Boston, MA USA; 9 Department of Human Physiology, Vidyasagar University, Midnapore, 721102 West Bengal India

**Keywords:** Betulinic acid analogue, EpCAM, Aptamer, Colorectal cancer, Tumor-microenvironment, Immune response

## Abstract

**Background:**

Betulinic acid (BA) has been well investigated for its antiproliferative and mitochondrial pathway-mediated apoptosis-inducing effects on various cancers. However, its poor solubility and off-target activity have limited its utility in clinical trials. Additionally, the immune modulatory role of betulinic acid analogue in the tumor microenvironment (TME) is largely unknown. Here, we designed a potential nanotherapy for colorectal cancer (CRC) with a lead betulinic acid analogue, named as **2c,** carrying a 1,2,3-triazole-moiety attached to BA through a linker, found more effective than BA for inhibiting CRC cell lines, and was chosen here for this investigation. Epithelial cell adhesion molecule (EpCAM) is highly overexpressed on the CRC cell membrane. A single-stranded short oligonucleotide sequence, aptamer (Apt), that folds into a 3D-defined architecture can be used as a targeting ligand for its specific binding to a target protein. EpCAM targeting aptamer was designed for site-specific homing of aptamer-conjugated-2c-loaded nanoparticles (Apt-2cNP) at the CRC tumor site to enhance therapeutic potential and reduce off-target toxicity in normal cells. We investigated the in vitro and in vivo therapeutic efficacy and anti-tumorigenic immune response of aptamer conjugated nanotherapy in CRC-TME.

**Methods:**

After the characterization of nanoengineered aptamer conjugated betulinic acid nanotherapy, we evaluated therapeutic efficacy, tumor targeting efficiency, and anti-tumorigenic immune response using cell-based assays and mouse and rat models.

**Results:**

We found that Apt-2cNP improved drug bioavailability, enhanced its biological half-life, improved antiproliferative activity, and minimized off-target cytotoxicity. Importantly, in an in vivo TME, Apt-2cNP showed promising signs of anti-tumorigenic immune response (increased mDC/pDC ratio, enhanced M1 macrophage population, and CD8 T-cells). Furthermore, in vivo upregulation of pro-apoptotic while downregulation of anti-apoptotic genes and significant healing efficacy on cancer tissue histopathology suggest that Apt-2cNP had predominantly greater therapeutic potential than the non-aptamer-conjugated nanoparticles and free drug. Moreover, we observed greater tumor accumulation of the radiolabeled Apt-2cNP by live imaging in the CRC rat model.

**Conclusions:**

Enhanced therapeutic efficacy and robust anti-tumorigenic immune response of Apt-2cNP in the CRC-TME are promising indicators of its potential as a prospective therapeutic agent for managing CRC. However, further studies are warranted.

**Graphical abstract:**

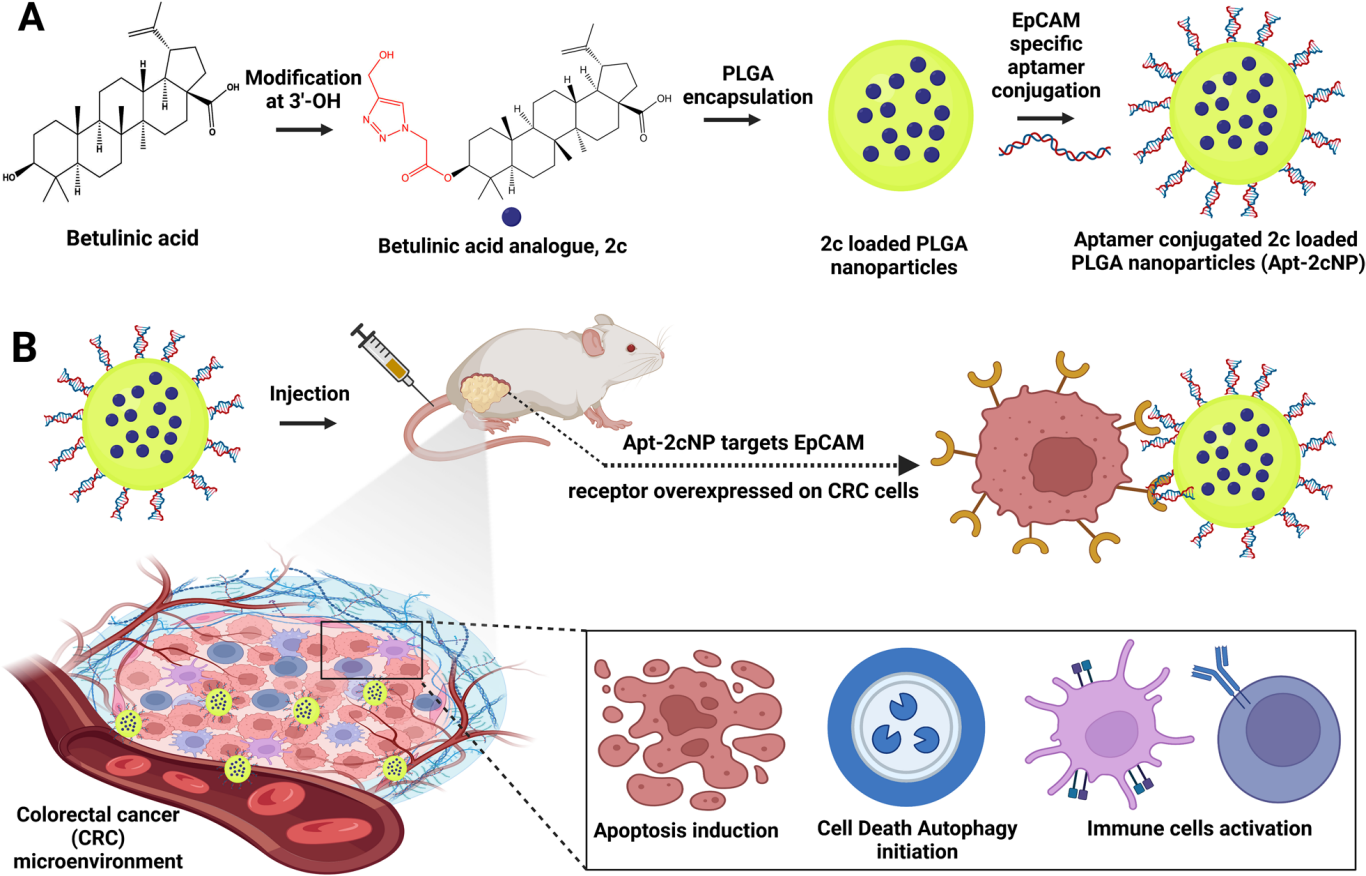

**Supplementary Information:**

The online version contains supplementary material available at 10.1186/s12929-024-01069-8.

## Background

Betulinic acid occurs in nature. It is a pentacyclic triterpenoid with anticancer, anti-inflammatory, and anti-HIV effects [1]. It prevents the interaction between topoisomerase enzyme and DNA and alters the membrane potential of mitochondria, leading to apoptosis through mitochondrial depolarization and DNA fragmentation [2]. BA also inhibits mitochondrial metabolism effectively in cancer cells [3]. However, the use of BA in the clinical management of cancer is limited due to its low solubility and potential off-target toxicities. Numerous derivatives of BA showed profound anticancer activities against many types of cancer [3–6]. BA analogue 2c, (3S)-3-[2-(4-hydroxymethyl-1H-1,2,3-triazol-1-yl)acetyloxy]-lup-20(29)-en-28-oic acid, is a modified triazole moiety attached to parent molecule through a linker, and a potent inducer of apoptosis in HT-29 human colorectal carcinoma cells [7]. However, the hydrophobic moiety had limited water solubility, poor bioavailability, and a short plasma half-life. These limitations can be surmounted when 2c is encapsulated in PLGA nanoparticles [8–10].

PLGA is one of the most attractive US-FDA-approved biodegradable and biocompatible polymers used for drug carriers and can be administered intravenously [11]. The polymeric encapsulation of BA or its analogue 2c with PLGA can enhance drug bioavailability, sustain drug release for a prolonged period, reduce drug loss, and potentiate enhanced permeability and retention (EPR) effects at leaky vasculature of tumor tissue [12]. Conjugating specific targeting ligands such as aptamers, peptides, and antibodies with the nanoparticle surface against the biomarkers overexpressed on tumor cells can direct them for site-specific drug delivery and minimize their off-target toxicities [13].

PLGA in the aqueous medium undergoes slow hydrolysis of its ester linkages [14]. PLGA biodegradation is a three-phase mechanism. Initially, polymeric chain scission occurs randomly, without much molecular weight loss, and with the formation of soluble monomers (lactic acid and glycolic acid) [15]. Subsequently, the next phase decreases polymeric molecular weight with a comparatively rapid mass loss, forming oligomers and soluble monomers [14]. In the third phase, oligomers change to soluble monomers that are ultimately eliminated in the form of carbon dioxide (CO_2_) and water (H_2_O) by the cellular citric acid cycle (Krebs cycle).

The advantage of aptamers over monoclonal antibodies is that aptamers do not show immunogenic reactions but have target-specificity similar to monoclonal antibodies. The aptamer selected for this study to target EpCAM receptor is a small single-stranded DNA (ssDNA) sequence that binds a large variety of EpCAM-positive carcinoma cells in vitro, including breast, ovarian, colon, prostate, and pancreatic carcinoma cells, but not to EpCAM-negative leukemia or lymphoma cells [16]. However, this aptamer has not been investigated for detailed therapeutic screening in vitro or in vivo for CRC. Furthermore, the aptamer has not been utilized as a drug-targeting ligand.

Hence, we designed aptamer conjugated nanoparticles for target-specific delivery of 2c to direct the substantial drug accumulation specifically to colorectal carcinoma tissue. We selected a phosphorothioate backbone-modified DNA aptamer (complete sequence provided in “Methods” section) that specifically binds with EpCAM receptors overexpressed on the surface of many solid tumor cells, including CRC cells [17]. Aptamers are one of the preeminence-targeting ligands. They are short single-stranded nucleotide sequences with a three-dimensional defined conformation that can efficiently bind to a target-specific antigen on the cancer cell surface. Aptamers are very stable at different pHs and at a broad range of temperatures and have a substantial affinity for target biomarkers on cancer cells [18]. Overall, this report is a comprehensive study on how aptamer conjugation potentiates the targeted therapy in preventing CRC growth and proliferation, along with the involvement of several key players of colorectal cancer signaling pathways by evaluating their pre- and post-treatment functional status. An additional focus on the nanoparticulated drug’s impact on in vivo immune response accomplished the overall therapeutic effect of Apt-2cNP in the CRC microenvironment.

## Methods

### Materials

Poly-(d,l-lactic *co*-glycolic acid) (PLGA; MW 4000–15,000; lactide to glycolide ratio 75:25) was purchased from Sigma Aldrich Chemicals Pvt. Ltd., Bangalore, India. Poly-(vinyl alcohol) (PVA, MW 125,000) was obtained from S.D. Fine-Chem. Ltd., Mumbai, India. Fluorescein isothiocyanate (FITC) and 3-(4,5-Dimethylthiazol-2-yl)-2,5-diphenyltetrazolium bromide (MTT) were purchased from Hi-Media Lab. Pvt. Ltd., Mumbai, India. Succinic acid, didodecyl dimethyl ammonium bromide (DDAB), and 1,2-dimethylhydrazine (DMH) were purchased from Sigma Aldrich (St. Louis, MO, USA). Dulbecco’s Modified Eagle Medium (DMEM) (High glucose), heat-inactivated fetal bovine serum (FBS), 4′,6-diamidino-2-phenylindole (DAPI) and 5,5′,6,6′-tetrachloro-1,1′,3,3′-tetraethyl benzimidazolylcarbocyanine iodide (JC-1) were purchased from Invitrogen (Carlsbad, CA, USA). All the chemicals and solvents used in the experiments were of analytical grade. Primers used in qPCR were purchased from Eurofins Scientific, Luxembourg. Live/Dead fixable aqua dead cell staining kit was purchased from ThermoFisher Scientific. Antibodies used in immunological analysis, such as fixable zombie-NIR, CD11c-APC, CD8a-FITC, CD317-PE, CD19-PE, MHCII-PerCP-Cy5.5, F4/80-APC, CD80-BV421, CD3-FITC, CD8-BV510, CD4-BV421, FOXP3-Alexafluor 647 were obtained from Biolegend. RORgT-PE was purchased from BD Biosciences. Alexa Fluor-594, anti-mouse IgG, was purchased from ThermoFisher Scientific, USA. ELISA kits for TNFα, IFNγ, and IL10 were purchased from Ray Biotech.

### Animal model development

Sprague–Dawley (SD) rats (body weight = 140–160 g) and Swiss albino mice (body weight = 20–25 g) of either sex (male:female = 1:1) were selected for the chemical-induced colorectal carcinoma model. The study plan was prepared as per the guidelines of the Committee for the Purpose of Control and Supervision of Experiments on Animals (CPCSEA), India (JU-AEC; Registration Number: 1805/GO/Re/S/15/CPCSEA) and was sanctioned by the Animal Ethics Committee (AEC), Jadavpur University, (Protocol approval. no.: AEC/PHARM/1701/3/2018). The animals were procured from the National Institute of Nutrition (Hyderabad, India) and were habituated in housing conditions of 25 °C ± 1 °C temperature, 55% ± 5% relative humidity, and normal day/night photoperiod for at least 2 weeks before conducting any experiment. For the development of CRC-bearing mice, Dimethylhydrazine (DMH) was injected intraperitoneally at a dose of 20 mg/kg body weight once a week for 12 weeks. In contrast, for generating CRC-bearing rats, DMH was administered intraperitoneally at a dose of 25 mg/kg body weight once a week for 9 weeks, as described previously [7].

Throughout the study period, the animals were nourished with standardized food and water. They were divided into five groups: normal (untreated) control mice, carcinogen (treated) control mice, carcinogen mice treated with 2c, carcinogen mice treated with 2cNP, and carcinogen mice treated with Apt-2cNP. Each group contained a minimum of five animals. Detailed information for each group is in Supplementary Table T1.

### Aptamer sequence

The aptamer sequence used in this study to target the EpCAM receptor was first developed by Zhao and Zhang [19] using SELEX technology. It is a small single-stranded DNA (ssDNA) sequence that binds a large variety of EpCAM-positive carcinoma cells in vitro, including breast, ovarian, colon, prostate, and pancreatic carcinoma cells, but not to EpCAM-negative leukemia or lymphoma cells [16, 17]. Most importantly, this aptamer has not yet been investigated for detailed therapeutic screening in vitro or in vivo for colorectal cancer.

For the current study, we modified the EpCAM targeting aptamer sequence with phosphorothioate backbone replacement to ensure that its plasma stability is retained. This aptamer conjugated nanoparticle, Apt-2cNP, has been extensively studied in a defined way and compared with an untargeted nanoparticle, 2cNP, to determine the potential effect of targeted therapy in preventing CRC growth and proliferation through in vitro and in vivo investigations.

The following 71-bp aptamer for the specific binding of EpCAM was synthesized on a 0.05 μmol scale and purified by high-performance liquid chromatography (HPLC) (synthesized by Eurofins Genomics India Pvt. Ltd., Bangalore, India). The aptamer sequence was selected because it is a highly explored aptamer with the lowest K_d_ value (12 nM) to EpCAM [16].

5′-A*T*C*C*A*G*A*G*T*G*A*C*G*C*A*G*C*A*T*G*C*G*G*C*A*C*A*C*A*C*T*T*C*T*A*T*C*T*T*T*G*C*G*G*A*A*C*T*C*C*T*G*C*G*G*C*T*C*T*G*G*A*C*A *C*G*G*T*G*G*C*T*T*A*G*T-3′-NH_2_

(* indicates the phosphorothioate backbone).

The 3′ end of the aptamer was modified by incorporating an amino group so that it could bind with the –COOH groups present on the PLGA nanoparticles by forming a peptide bond. Additionally, the aptamer was conjugated with Cy5 to make the nanoparticles visible under a fluorescence microscope and detected by flow cytometer (FACS).

## Formulation development, physical and mechanistic overview for the study

### Preparation of BA analogue, 2c-loaded PLGA nanoparticles (2cNP)

Briefly, 5 mg of **2c** and 50 mg of PLGA (75:25) were dissolved in 3 ml of organic solvent mixture [Dichloromethane (DCM): Acetone:: 4:1 v/v]. This solution was emulsified with 1.5 ml of 2.5% aqueous PVA by homogenizing at 20,000 rpm for 3 min using a high-speed homogenizer (IKA Laboratory Equipment, Model T10B, Ultra-Turrax, Staufen, Germany). The emulsion thus obtained was further emulsified with 75 ml of 1.5% aqueous PVA solution by homogenizing at 20,000 rpm for about 10 min to get a w/o/w type double emulsion [15]. Size reduction of the emulsion droplets was carried out by sonication in a bath-sonicator for 30 min. The final double emulsion was then stirred overnight on a magnetic stirrer at room temperature for the removal of organic solvents and the subsequent formation of polymeric nanoparticles. The nanoparticles were then separated from the dispersion by centrifugation: once at 5000 rpm for 10 min, followed by centrifugation of the supernatant at 16,000 rpm for 45 min. Separated nanoparticles were washed twice with double distilled water to remove excess PVA attached to them and finally lyophilized in a freeze dryer (Laboratory Freeze Dryer, Instrumentation India, Kolkata, India) to get dry powder.

### Conjugation of aptamer on the surface of nanoparticles

2cNP was first suspended in a 5 mg/mL concentration in deionized water and then agitated with 200 mM of EDC and 100 mM of NHS for 30 min at 25 °C. The resulting 2cNP with activated COOH groups was then washed with DNase-RNase free water to remove excess EDC/NHS [16, 17]. A portion of 0.5 mg/mL of aptamer solution was allowed to denature–renature by keeping it at 85 °C for 10 min, followed by cooling in an ice-water bath for 10 min. The activated 2cNP dispersion was then mixed with the denatured–renatured aptamer and allowed to react for at least 6 h with slow rotation. The covalently linked Apt-2cNP was finally rinsed with deionized water [18].

### Confirmation of aptamer conjugation by agarose gel electrophoresis and XPS analysis

Confirming the successful conjugation of aptamers to nanoparticles is a crucial step in aptamer-based drug carriers. The aptamer conjugation on 2cNP nanoparticles was confirmed by agarose gel electrophoresis. Samples were loaded inside the well of the gel in the following sequence: DNA marker, 2cNP, free aptamer, Apt-2cNP. Ethidium bromide (0.5 mg/mL) was added to visualize nucleic acids. The electrophoresis was carried out at 50 V for 160 min.

XPS is a surface-sensitive analytical technique that quantifies the elemental composition and chemical state of a material’s surface by analyzing the kinetic energy and binding energy of emitted photoelectrons. X-ray photoelectron spectroscopy (XPS) analysis was carried out for 2c, 2cNP, and Apt-2cNP in an Omicron Multiprobe Electron Spectroscopy System using a monochromated X-ray of 200 eV emitting from an AI Kα type source gun [20].

### Analysis of nanoparticle morphology

The analysis of nanoparticles’ morphology, encompassing their shape, size, and structure, holds significant importance across diverse fields such as materials science, catalysis, and biotechnology. Field Emission Scanning Electron Microscopy (FESEM) stands out as a widely utilized and potent technique for this purpose. The morphology of dry nanoparticles after aptamer conjugation was observed using field emission scanning electron microscopy. An appropriate amount of Apt-2cNP was mounted on carbon tape, attached with a metal stub, and coated with gold using a sputter coater (around 4 nm layer thickness), and observed under a field emission scanning electron microscope (JEOL JSM-7600F, Japan) at 20,000 × magnification. Atomic Force Microscopy (AFM) serves as a potent tool for examining nanoparticle morphology, offering high-resolution topographical details and precise measurements of particle size, shape, and distribution. Atomic force microscopy was utilized to observe the surface morphology of aptamer-conjugated nanoparticles in wet conditions. Dry nanoparticle powder (Apt-2cNP) was dispersed in MilliQ water at a concentration of 100 μg/ml with the help of brief sonication and vortexing. The suspension of nanoparticles was then filtered with a 0.22 μm filter to eliminate any preformed large particles. 5 μl of this filtered dispersion was placed on a freshly cleaved mica sheet and allowed to air dry at room temperature. After evaporating water, the thin clear layer formed on the mica sheet was examined under an AFM instrument (5500 Agilent Technologies, Santa Clara, CA, USA) in search of nanoparticles. The scanning was carried out under ambient conditions in tapping mode using a silicon nitride probe having a resonance frequency and a force constant of 150–350 kHz and 0.4 N/m, respectively. The images were captured over a 9 μm × 9 μm and were analyzed with the help of Agilent Pico View software (version 1.12). Cryo-Transmission Electron Microscopy (Cryo-TEM) emerges as another powerful method for scrutinizing the morphology of nanoparticles in their natural state, furnishing detailed structural data at high resolutions without the necessity of staining or desiccating the sample [20, 21]. The internal structure of Apt-2cNP was analyzed using cryo-transmission electron microscopy. Apt-2cNP was dispersed in MilliQ water to attain approximately a 50 μg/ml concentration, and 5 μl from this solution was mounted on a 300 mesh carbon-coated copper grid (previously glow discharged to minimize charge interaction). Then, it was plunge-frozen in liquid ethane, and excess liquid was removed with specially designed filter paper using a Vitrobot instrument (FEI Company, USA). The copper grid was then transferred to a cryoholder and observed in a Technai POLARA (FEI Company, Oregon, USA) electron microscope, empowered by a 300 kV field emission gun (FEG). Images were captured in a 4 k × 4 k charge-coupled device (CCD) camera.

### Determination of drug loading (%) and entrapment efficiency (%)

The drug (2c) was extracted from 2 mg of Apt-2cNP to determine the drug loading by dissolving in 2 ml of a solvent mixture of acetonitrile: water:: 85:15 (v/v) using an incubator shaker for 4 h [16]. The insoluble fraction of nanoparticles was separated by cold centrifugation at 16,000 rpm for 15 min, and the clear supernatant was collected for spectrophotometric analysis at the corresponding λ_max_ of 340 nm. The concentration of the drug was calculated from a prepared calibration curve.

Drug loading was estimated using the following equation:

1 $${\%}\,\text{Drug\, loading }(\text{actual}) =\frac{\text{ Amount of drug in nanoparticles }}{\text{Amount of nanoparticles obtained}}\times 100.$$

Entrapment efficiency was calculated using the following equation:

2 $${\%}\,{\text{Entrapment efficiency}}=\frac{\text{Drug loading }(\text{actual}) ({\%})}{\text{Drug loading }(\text{theoretical}) ({\%})}\times 100.$$

### In vitro drug release study

The evaluation of in vitro drug release is essential for assessing the efficacy of nanoparticle-based drug delivery systems and comprehending their drug release kinetics. These studies yield valuable insights into the rate and extent of drug release from nanoparticles under controlled conditions, thus facilitating predictions regarding their in vivo behavior.

Drug release behavior from Apt-2cNP was studied in phosphate-buffer saline (PBS), in PBS containing 0.5% β-cyclodextrin (β-CD), and in sodium acetate buffer of pH 5.5. 2 ml of Apt-2cNP suspension (1 mg/ml) in respective buffer was placed in an Eppendorf tube and was agitated in an incubator shaker (Somax Incubator Shaker; ShenjhenPango Electronic Co. Ltd., Shenzhen, China) at 37 °C [13, 22]. 1 ml of suspension was withdrawn from this vial at different time intervals (0.5 h, 1 h, 2 h, 4 h, 6 h, 8 h, 10 h, 12 h, 24 h, 48 h, 72 h, 168 h, 336 h, 504 h, 672 h, 840 h, 1008 h, 1128 h, 1344 h, 1512 h), centrifuged and the supernatants were analyzed for drug content using UV–visible spectroscopy. The pellet obtained on each centrifugation was redispersed by adding 1 ml of fresh PBS (containing 0.5% β-cyclodextrin) for every withdrawal. A cumulative % drug release vs time curve was generated using a calibration curve. The kinetics of drug release from Apt-2cNP were studied following different kinetic models such as zero order, first order, Higuchi, Hixon-Crowell, and Korsmeyer-Peppas models.

### Molecular docking analysis

Molecular docking analysis is a computational technique used in structural bioinformatics and molecular modeling to predict the preferred orientation and binding affinity of a small molecule (ligand) when bound to a target macromolecule (receptor), such as a protein or nucleic acid. This method plays a crucial role in drug discovery, lead optimization, and understanding molecular interactions at the atomic level. The X-ray crystallographic structures of topoisomerase-I and topoisomerase-II enzymes were downloaded from the RCSB website, and their pdb codes are 1A36 and 2RGR, respectively [21, 22]. The energy-minimized structures of BA and 2c were obtained from  Density functional theory (DFT) calculations with B3LYP functional and 6–311 g basis sets using Gaussian 09W [23]. The same method was used for the molecular electrostatic potential map calculations. These structures were utilized in the docking calculations. The Genetic-Lamarckian algorithm was used for the docking calculations with the help of AutoDock4.2 [24] using a 60 × 60 × 60 grid box. Chimera 1.10.2 [25] was used for the visualization of docking results.

To conduct the molecular docking investigation, we chose the receptor as the epithelial cell adhesion molecule to which the aptamer can bind precisely in silico. In this study, “in-silico” predictions give an understanding of the binding capability of the compound (**2c**) to the topoisomerase proteins. The H-bond formation and the hydrophobic interaction ability of betulinic acid derivative (**2c**) suggest its preferentially binding with TIase-II over BA (a well-known TIase-II inhibitor). The predictions support the factual findings of the manuscript. We have found that **2c** inhibited the function of topoisomerase II enzymes (TOP2A and TOP 2B), leading to DNA chain-break and cell cycle arrest, inducing cancer cell death.

The crystal structure of the EpCAM, 4MZV, was received from the RCSB protein data bank, and the DNA aptamer structure was generated in the Discovery studio visualizer 2021 by inserting the 71 bp DNA sequence and converting it to PDB format. Initially, the receptor was prepared by removing water molecules and replacing them with polar hydrogen atoms and charges. The ligand molecule was then prepared for docking analysis utilizing Discovery Studio Visualizer 2021. Further interactions between the aptamer and EPCAM were investigated using the HDOCK blind docking service (http://hdock.phys.hust.edu.cn/) [26]. In the docking procedure, the initial step was to provide inputs for the receptor and ligand. The docking score assesses the quality of a projected protein-nucleotide binding mechanism. The docking study was carried out using the Discovery Studio 2021 Client Program. The interactions were depicted with the help of Biovia Discovery Studio 2021 [27].

### MTT assay

The MTT assay measures toxicity response against a treatment to a cell, and cell viability. It is widely employed in various fields, including cell biology, cancer research, and drug development studies. The cytotoxic effect of free drug (2c), 2cNP, and Apt-2cNP in colorectal cancer cell lines was evaluated on HT-29 and HCT-116 cell lines. After 48 h of incubation, the experimental procedure and quantitative data analysis were conducted using the previously reported method [7]. The detailed methodology is described in the supplementary file.

### In vitro cellular uptake study

An in vitro cellular uptake study evaluates the cellular internalization and accumulation of various molecules, nanoparticles, or other materials under controlled laboratory conditions. Cellular uptake of Cy5 conjugated nanoparticles was quantified by flow cytometer (BD LSR Fortessa, BD Biosciences) using the channel for Cy5 (Excitation/ Emission 645 nm/664 nm). The data was analyzed using FACS Diva software [28]. Additionally, the cells were observed under a confocal laser microscope (Olympus FluoView FV10i, Olympus) using the filters for Cy5 (Excitation/ Emission 645 nm/664 nm) and DAPI (Excitation /Emission 359 nm/461 nm) [29, 30].

### Apoptosis assay

An apoptosis assay detects and quantifies programmed cell death (apoptosis) in cell populations. It demonstrates the cellular death process through various treatments to understand treatment efficacies in cancer cell elimination. We monitored the cell death induction mechanisms in colorectal cancer cells after treatment with 2c, 2cNP, and Apt-2cNP for 24 h and 48 h. HT-29 and HCT116 cells were seeded in 60 mm tissue culture dishes at a density of 1 × 10^6^ per dish and incubated overnight in a humidified incubator at 37 °C. After that, the cells were treated with 2c, 2cNP, and Apt-2cNP with respective IC_50_ concentrations for 24 h and 48 h to evaluate apoptotic cell death induction. Two parallel groups were run with media alone to provide the control and the 'unstained' group (the cells that were untreated and were not stained with any dye during analysis). After completion of treatment, the supernatant was removed, and the cells were carefully washed with PBS to remove residuals from the formulations. The cells were then detached through trypsinization and redispersed in 1 × binding buffer after washing with PBS (pH 7.4). 5 µl of Annexin V-FITC (BD Bioscience) was added to this 100 µl cell suspension and incubated for 20 min in the dark [31]. After that, the cells were diluted with an additional 400 µl binding buffer (1 ×), and 5 µl of propidium iodide solution (1 mg/ml stock) was added to the cells in a light-protected condition before acquisition in FACS. The cells were then transferred to a FACS tube and analyzed in a flow cytometer (BD LSR Fortessa, BD Bioscience) to determine the proportions of live, dead and apoptotic cells.

### Determination of mitochondrial membrane depolarization

Determining mitochondrial membrane depolarization assesses mitochondrial functional status and apoptosis induction potential or cellular stress. A loss or disruption of membrane potential is often associated with mitochondrial dysfunction and can trigger apoptotic signaling pathways. Mitochondrial membrane depolarization is a key marker of apoptosis induction. Thus, we followed the standard method for measuring mitochondrial membrane potential using FACS by fluorescence emission after JC-1 staining. Briefly, 1 × 10^6^ HCT116 and HT-29 cells were seeded in 60 mm dishes, incubated overnight in a humidified incubator, and then treated with 2c, 2cNP, and Apt-2cNP for 24 h and 48 h. Thereafter, the cells were removed from the dishes and incubated with 10 μl of 200 μM JC-1 in 1 ml complete media for 10 min at 37 °C in the dark. The media was replaced by PBS (pH 7.4) through centrifugation, and the cells were analyzed in a FACS instrument.

### Cell cycle analysis

Cell cycle analysis evaluates cell distribution patterns in the cell cycle-phases. This analysis is crucial for understanding cellular proliferation, growth, and response to various treatments or conditions. It is widely employed in fields such as cell biology, cancer research, and drug development. Next, we performed a cell cycle analysis to estimate the percentage of cell population in different stages of cell cycles, G0/G1, S, and G2/M. HT-29 and HCT116 cells were plated in 60 mm cell culture dishes (1 × 10^6^ per dish) and allowed to grow overnight in DMEM medium at 37 °C. Then, the cells were treated with 2c, 2cNP, and Apt-2cNP (at respective IC_50_ concentrations) for 24 h and 48 h. After completion of incubation timing, the treatment solution was removed, and the cells were stained alive with Hoechst 33,258 (5 µg/ml final concentration) [32]. The stained cells were then analyzed in a flow cytometer (BD LSRFortessa, BD, CA, USA) to assess the population of cells at different stages of the cell cycle.

### Colony formation assay

The colony formation assay, also known as the clonogenic assay, is a cell biology technique used to evaluate the ability of individual cells to undergo unlimited division and form colonies. This assay is commonly employed in cancer research, radiation biology, and drug screening studies to assess cells' proliferative potential and sensitivity to various treatments or conditions. A clonogenic assay was conducted here to evaluate the inhibitory effect of the free drug and nanoencapsulated therapies on the tumor-initiating capacity or colony formation potential of the colorectal cancer cells. About 500 cells were seeded for both the cell lines HT-29 and HCT116 in each well of a 12-well plate, followed by incubation overnight. The cells were treated with 2c, 2cNP, and Apt-2cNP every 3 days for a total of 12–14 days [33, 34] except for the control groups. After completion of drug incubation, the treated medium was withdrawn, and the cells were washed with PBS. 10% acetic acid (in methanol) was used as a fixation buffer, and colonies were stained with crystal violet solution (0.5% crystal violet in 25% methanol) and washed with PBS three times to remove excess dye. After air-drying overnight, the next day, the images of colonies were captured, and colony counting was taken. After imaging, the crystal violet stained colonies were dissolved in 10% acetic acid solution for 15 min under shaking conditions, and the absorbance of the supernatant was detected using a plate reader at 510 nm.

### Autophagy detection by acridine orange staining

Autophagy is a cellular process in which the damaged or unnecessary components are degraded and recycled by the cell. Its detection is important in various fields, including cell biology, cancer research, and neurodegenerative disease studies. One commonly used method for detecting autophagy is acridine orange staining. Induction of autophagy in HT-29 and HCT116 cells in the different treatment groups was evaluated using acridine orange staining. Briefly, 2 × 10^4^ cells were seeded on a coverslip, placed into a 35 mm tissue culture dish, and incubated overnight in a 37 °C incubator. The cells were then treated with 2c, 2cNP, and Apt-2cNP at their respective IC_50_ concentrations for 24 h and 48 h. After removing the treatment media, the cells were stained with acridine orange (6 µg/ml, 3 min). The coverslips placed onto the slides were observed under a confocal laser microscope (Zeiss LSM 900) using green (excitation/emission at 500 nm/526 nm) and red (excitation/ emission at 400 nm/650 nm) filters. The dual color images were obtained by merging the images obtained from green and red filters using Zeiss proprietary software Zen Lite 2.0.

### Autophagic flux detection by Cyto ID Green fluorescent probe

The detection of autophagic flux using the Cyto-ID Green fluorescent probe monitors autophagy, a cellular process of cytoplasmic component degradation and recycling. The technique visualizes and quantifies autophagic vacuoles, which are crucial components of the autophagic pathway. Briefly, 1 × 10^6^ HT-29 and HCT116 cells were seeded in each well of 6-well cell culture plates and incubated overnight at 37 °C incubator. Thereafter, the cells were treated with 2c, 2cNP Apt-2cNP (at its IC_50_ concentration) for 24 h and 48 h. After completion of treatment, the media was removed, followed by washing with PBS. The cells were stained with the Cyto ID Green fluorescent probe (Enzo Life Sciences, Farmingdale, NY,USA), following the manufacturer's guidelines [35]. Finally, the stained cells were analyzed using a flow cytometer (BD LSR Fortessa, BD Bioscience) to estimate autophagic flux formation quantitatively.

### Hemolysis study

Hemolytic activity of a formulation indicates the safety issue for its intravenous use. Low hemolytic value supports the formulation to be safe while in blood. To test the hemocompatibility of the BA analogue, 2c, and its different nanoformulations (2cNP and Apt-2cNP) at various concentrations, blood was collected from male Swiss albino mice and placed in heparinized tubes and centrifuged at 4 °C for 5 min at 1000*g*. The erythrocytes were then washed three times with PBS, pH 7.4. The cells were suspended at a concentration of 2% in PBS, pH 7.4. A 96-well plate was prepared with 190 µL of the cell suspension in each well. 2cNP and Apt-2cNP nanoparticles were added to each well in varying concentrations of 2c, ranging from 10 to 100 µM. After incubating for two hours at 37 °C with gentle stirring, the un-lysed erythrocytes were separated by centrifugation at 10,000*g* for 5 min. The percentage of hemolysis was determined by measuring the optical density (OD) of the supernatant at 570 nm and using the procedure to compute the absorbance factor of a sample that was 100% hemolytic [36, 37].

$$\text{Hemolysis }({\%})=\frac{\text{Abs}-\text{Abs}_0}{\text{Abs}_{100}-\text{Abs}_0} \times 100.$$

Abs, Abs_0_, and Abs_100_ represent the absorbance of samples, a solution with 0% and a solution with 100% hemolysis, respectively.

### Analysis of gene expression by qPCR

Gene expression analysis by quantitative real-time polymerase chain reaction (qPCR) is widely used in molecular biology and genetics research. qPCR allows the detection and quantification of specific RNA transcripts, providing insights into the expression levels of genes under various conditions or in different cell types or tissues. The expression of several genes related to apoptosis, autophagy, DNA synthesis, and inflammatory pathways were analyzed after treatment with 2c, 2cNP, and Apt-2cNP to understand the molecular mechanism behind their cytotoxic effects and to determine differences between aptamer-conjugated and unconjugated nanoparticles. Total RNA from CRC cancer tissue before and after treatment was isolated after completion of treatment time using TRIzol, following the manufacturer’s protocol. The RNA was checked for quality by measuring the A_230/280_ ratio and quantified in a NanoDrop instrument. Then, cDNAs were prepared using RevertAid First Strand cDNA synthesis kit (ThermoFisher) followed by amplified using SYBR Green Master Mix (BioRad) in a thermal cycler (Veriti 96-well, Applied Biosystems, Massachusetts, USA). Relative transcript abundance values obtained from the instrument were plotted in bar diagrams. A total of 12 genes (caspase 3, Bcl-2, Bcl-XL, Bax, Atg5, LC3B, Beclin, p62, NF-κB, APC, p53, and TOP2) were analyzed for each sample. Sequences of the primers used (both forward primer and reverse primer) for each gene are in Supplementary Table T2.

### Evaluation of inflammatory response in tumor microenvironment

Colorectal tissues were isolated from the CRC-bearing mice before and after treatment with 2c, 2cNP, and Apt-2cNP, followed by washing with ice-cold PBS. Colons were then minced into small pieces and agitated with predigestion buffer (HBSS, 10 mM HEPES, 5 mM EDTA, 1 mM DTT, 5% FBS) for 20 min in a 37 °C incubator-shaker. The single cells suspension from the colonic tissues was prepared using a 100 µm cell strainer. The remaining tissue pieces collected from the cell strainer were then cut into finer fragments and treated with digestion solution (HBSS, 10 mM EDTA, 0.5 mg/ml collagenase D, 3 mg/ml dispase II and 5% FBS) in a 37 °C incubator-shaker for 30 min. The dissociated cells from the tissues were collected again through a 100 µm cell strainer. All the single-cell suspensions were pooled together and centrifuged using Percoll gradient. The lymphocytes were suspended in 4 ml of 40% Percoll solution and layered over 12 ml of 80% Percoll solution. Centrifugation was then carried out without a break at 1000* g* for 20 min. The T cells were collected from the liquid interphase and resuspended in PBS or RPMI 1640 medium for further experiments.

To analyze cytokine levels (TNFα, IFNγ, and IL-10) and all other ELISA assays, sandwich ELISA kits were used. 100 μl of cell culture supernatant were incubated in each ELISA plate well for 2.5 h at room temperature (RT). Incubation was done with 100 μL of biotin antibody (60 min at RT) followed by 100 μL of streptavidin solution (60 min at RT). Each step was performed following gentle washing of the wells with PBS. 100 μl of TMB one-step substrate reagent was added to each well and incubated for 30 min at RT. The reading was monitored using a multi-plate reader and then compared with the standard curve for actual measurement of the cytokine level.

### Flow cytometric analysis of immune cells in CRC murine tissue samples

The tumor microenvironment (TME) in colorectal cancer (CRC) is characterized by a complex interplay of various immune cells that can promote or inhibit tumor growth. Understanding the roles and interactions of these immune cells is crucial for developing effective immunotherapies. Different mice groups were sacrificed after treatment, and colorectal cells were harvested. The single-cell suspension of the cells is done by overnight enzymatic degradation and passing through cell strainers. The cells were washed with phosphate buffer saline (PBS) and centrifuged at 1000 rpm for 10 min at 4 °C, and the supernatant was discarded. The cell pellet was then resuspended in 2 ml RBC lysis buffer to get rid of the RBC in the solution and then incubated for 5 min at RT. The cells were then centrifuged for 10 min at 4 °C at 1000 rpm, and the pellet was resuspended in PBS for washing. The washing was repeated two times. The cells were finally resuspended in 2% FBS in PBS. The cells were first blocked by incubating with anti-CD16/CD32 and washed two times with PBS.

The cells were then aliquoted and incubated with different fluorochrome-conjugated antibody combinations from Biolegend unless otherwise mentioned, following the manufacturer’s protocol for antibody dilution, incubation duration, and washing steps. For intracellular staining, fixation was done by using 4% paraformaldehyde, followed by the permeabilization of the cells with the 0.1–0.3% Triton™ X-100 and two times washing with PBS.

Additionally, we monitored autophagosomes in single-cell suspension of epithelial cells from CRC mice tumor tissue using fluorescent probe Cyto-ID Green staining by flow cytometric analysis. Fluorescence signals from the labeled cells were acquired using the FACS machine and analyzed using FlowJo software.

### Analysis of protein expression by Western blotting

Western blotting is a widely used analytical technique in molecular biology and biochemistry to detect and quantify specific proteins in a complex mixture of proteins from cell or tissue extracts. Treated (with 2c, 2cNP, and Apt-2cNP) and untreated (control) HT-29 and HCT-116 cells were lysed using RIPA lysis buffer (Sigma Aldrich, USA). Each sample was then centrifuged at 16,000*g* for 20 min, and the clear supernatant was collected. Protein content in the supernatant was determined using a Bradford reagent. 20 μg protein/lane was loaded on an SDS-PAGE gel (10%), followed by electro-transferred onto a PVDF membrane. The membrane was blocked by 5% skimmed milk in Tris-buffered-saline-tween (TBST) solution (20 mM Tris–HCl, pH 7.4, 150 mM NaCl, 0.02% Tween 20) for 1 h followed by probing with primary antibodies (Topoisomerase-II/Beta-actin) for overnight at 4 °C. After washing 3 times with TBST, the membranes were incubated with secondary antibody for 1 h. Finally, the membrane was washed again 3 times with TBST and visualized by chemiluminescence kit using a Gel Doc [38].

### In vivo colonic distribution of nanoparticles in CRC-bearing Swiss albino mice

The evaluation of in vivo colonic distribution of nanoparticles in colorectal cancer (CRC)-bearing Swiss albino mice is a critical study that can provide valuable insights into the potential of nanoparticle-based drug delivery systems for targeted therapy against colorectal cancer. We injected Cy-5 dye-loaded 2cNP and Apt-2cNP into CRC-bearing Swiss albino mice through the tail vein (25 mg/kg) and sacrificed the animals after 24 h and 48 h. The colons were isolated from the animals, fixed with 4% formaldehyde solution, and sectioned with a microtome of their paraffin blocks. The sections (approximate thickness 5 µm) were mounted on microscopic slides. Then, the sections were stained with DAPI (300 nm) to detect the nucleus. The colon sections were then observed under a confocal laser microscope (Olympus Fluoview 10i, Olympus) using the channels of Cy5 (Excitation/Emission 645 nm/ 664 nm) and DAPI (Excitation/ Emission 359 nm/ 461 nm.

### In vivo antitumor efficacy of free drug and nanoparticles with the histopathological observations

Evaluating in vivo antitumor efficacy with histopathological observations is essential to preclinical studies in cancer research and drug development. This approach combines the assessment of tumor growth inhibition with the histological examination of tumor tissues to provide insights into the mechanisms of action and potential side effects of investigational anticancer agents. DMH-induced CRC was developed in Swiss albino mice and Sprague Dawley rats following the method described in an earlier section. After histological confirmation of CRC development, the mice were treated with 2c, 2cNP, and Apt-2cNP at 300 mg/kg intraperitoneally once a week for 9 weeks. After that, the animals were sacrificed, and their colons were isolated and sectioned for microscopic slides, followed by histological analysis carried out through H&E staining and Ki67 immunohistochemistry assay. H&E staining was additionally carried out for the liver and kidneys, which were also isolated from normal mice, CRC-bearing mice, and CRC-bearing mice after treatment with 2c, 2cNP, and Apt-2cNP. Ki67-positive and -negative cells were counted in microscopic images taken after staining using QuPath-0.3.2 software.

### Pharmacokinetic evaluation

Pharmacokinetic evaluation informs the time course of drug absorption, distribution, metabolism, and elimination (ADME) within the body. It is a crucial aspect of drug development and is vital in understanding how a drug behaves in vivo. 2c, 2cNP, and Apt-2cNP were injected separately into CRC-bearing mice through intravenous injection (dose: 25 mg/kg). Blood samples were withdrawn at 4 h, 8 h, 10 h, 12 h, 24 h, 48 h, 72 h, and 96 h post-injection, and plasma concentrations of 2c for all samples were determined using LC–MS following a previously described method [39]. A plasma concentration vs time curve was generated using GraphPad Prism (version 5.0). Cmax and Tmax were determined directly from the curve. AUC, AUMC, and AUC_0–∞_ were calculated using the trapezoidal method. t_1/2_, MRT, and clearance values were calculated using standard formulas.

### In vivo biodistribution in CRC-bearing Sprague Dawley (SD) rats

In vivo biodistribution studies in CRC-bearing Sprague Dawley (SD) rats are critical for understanding therapeutic agents' pharmacokinetics and tissue distribution, which can inform their efficacy and safety profiles. These studies involve tracking the distribution of a drug or therapeutic compound throughout the body after administration. The in vivo biodistribution study was carried out in CRC-bearing SD rats (n = 5). 2cNP and Apt-2cNP were radiolabeled with ^99m^Tc following the previously described method [40]. The radiolabeled nanoparticles were injected through the tail vein in experimental animals. The animals were hydrated well with intraperitoneal administration of normal saline (0.9%) prior to administering the intravenous injection. The radioactivity injected into the rat was 14–18 MBq/Kg body weight. 1 h, 2 h, and 5 h after injection, the animals were sacrificed, and different organs (liver, kidney, colon, lung, heart, stomach, muscle) and blood and urine samples were collected in pre-weighed counting vials. The radioactivity present in each organ and fluid sample was measured using a gamma scintillation counter (Electronics Corporation of India Limited, Hyderabad, India). The data were calculated as percent injected dose per g (% ID per g) of tissue or organ following the equation (Eq. 3).

3 $${\%ID }=\frac{Radioactivity\,present\, in\, an\, organ }{Total\, radioactivity\, injected\, in\, blood} \times 100.$$

### Gamma scintigraphic imaging using ^99m^Tc radiolabeled Apt-2cNP

Gamma scintigraphic imaging using ^99*m*^Tc (Technetium-99m) radiolabeled nanoparticles is a powerful technique for visualizing the biodistribution and accumulation of nanoparticles in vivo [41]. This method combines the advantages of nanoparticle-based drug delivery with the sensitivity and resolution of gamma scintigraphy, allowing for detailed tracking of the nanoparticles in a living organism. Scintigraphic imaging was carried out in CRC-bearing SD rats to visualize the in vivo distribution of 2cNP and Apt-2cNP after intravenous injection. 2cNP and Apt-2cNP were radiolabeled using ^99m^Tc following the method as described earlier [7, 42]. The radiolabeled nanoparticles were injected into CRC-bearing SD rats through the tail vein (containing 15–20 MBq/Kg body weight), and the radio images were captured at 1 h, 2 h, and 5 h post-injection in a gamma-scintigraphy camera (GE Infinia, GE Healthcare, India).

### Statistical analysis

Each of the experiments, except as otherwise stated, was executed at least three times, and the corresponding data provided the mean value along with the standard deviation of the means. Statistical analysis was performed using Student’s t-test, one-way ANOVA followed by Tukey’s post hoc test, and two-way ANOVA analysis, which was tested by Bonferoni’s post-test. Images, bar diagrams, and graphical representations were generated using Graph Pad Prism software, Gimp 2.10.30, ImageJ, Flow Jo, AutoDock 4.2, and Origin 2021. Schematic representation was created using Biorender.com. Probability value, p < 0.05, was considered as the statistical level of significance.

## Results

### Physicochemical characterization of experimental naoparticles

**Fig. 1:**
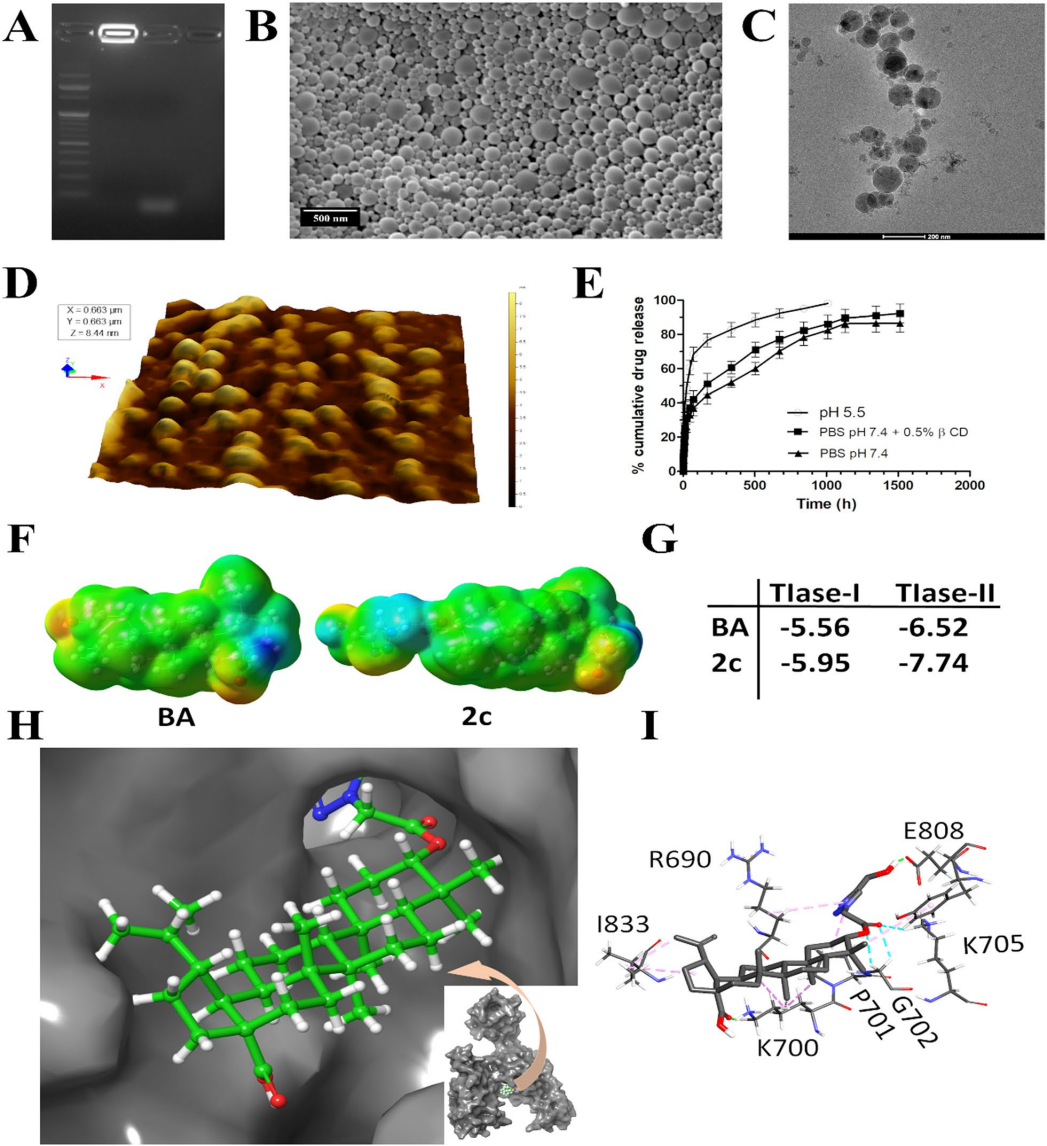
Particle characterization and determination of in silico mechanism of action. **A** Gel electrophoresis demonstrating conjugation of the aptamer to the nanoparticle**.** 1st well: standard DNA ladder; 2nd well: Apt-2cNP; 3rd well: free aptamer; 4th well: 2cNP. **B** Field emission scanning electron microscopic image of Apt-2cNP. **C** Transmission electron microscopic image of Apt-2cNP. **D** Atomic force microscopic image of Apt-2cNP. **E** Drug release profile of Apt-2cNP in PBS (pH 7.4), in PBS, containing 0.5% β-cyclodextrin, and in sodium acetate buffer of pH 5.5. Data shows mean ± SD (n = 3). **F** Molecular electrostatic potential map of BA and its analogue 2c. The blue, red, and green color indicates the most electron-deficient (hydrogen bond acceptor), -rich (hydrogen bond donor), and -neutral (hydrophobic) regions, respectively. **G** The change of Gibbs free energy values (kCal/mol) for the interactions of BA and 2c with topoisomerase-I (TIase I) and topoisomerase-II (TIase II) enzymes separately. **H** Protein pocket model representing the docking of compound 2c at the active site of topoisomerase-II. **I** Docking represented in a stick model (3D) shows the interaction of 2c with different amino acid residues at the active site of topoisomerase-II

We first characterized the aptamer-conjugated (Fig. 1A) and non-conjugated nanoparticles physicochemically. The drug loading (%) of Apt-2cNP (7.9 ± 0.2%) was almost similar to 2cNP (8.0 ± 0.5%). The entrapment efficiency of Apt-2cNP was 87%, which indicates that the nanoparticles synthesis process successfully entrapped a good amount of drug with a little drug loss. Using dynamic light scattering (DLS), we found that the average hydrodynamic diameter of Apt-2cNP was 188 ± 14 nm, whereas, for 2cNP, the average hydrodynamic diameter was 180 ± 15 nm. A very little size enhancement was seen due to aptamer incorporation to the nanoparticle surface. The average zeta potential of Apt-2cNP and 2cNP was − 11.5 ± 0.5 mV and − 7.94 ± 1 mV, respectively (Supplementary Table T3). Aptamer conjugation showed enhancement of zeta potential. The zeta potential values suggest that the nanoparticles were stable under the suspended conditions in water and can be stored as a freeze-dried powder and to be dispersed in water before administration [17].

Using FESEM, we found that Apt-2cNPs had a smooth exterior surface, without any crack or deformation (Fig. 1B), having a size range of around 180–188 nm (results obtained from DLS, Supplementary Table T3). However, there were many particles of around 100 nm in size (Fig. 1B). The nanoparticles were thickly distributed. The presence of some comparatively larger particles seemed to enhance the average particle size. The TEM image showed that drug particles were scatterly distributed within the nanoparticles (Fig. 1C). The nanoparticles in TEM analysis also showed a size below 200 nm. The Atomic force microscopy (AFM) confirmed that particles were in nanodimension with smooth surfaces (Fig. 1D).

### Molecular docking and X-ray photoelectron spectroscopic analysis

A molecular docking study confirmed the affinity or interactions between the 71 bp DNA aptamer with the EpCAM receptor. The results indicate that the aptamer's nucleotide bases efficiently bind to diverse amino acid residues of the receptor (Supplementary Figure S1, Supplementary Table T4). The DNA aptamer binds to the EpCAM through various amino acid residues which include serine (Ser60, Ser199), aspartic acid (Asp100, Asp177, Asp206), glutamine (Gln175), arginine (Arg138, Arg140), glutamic acid (Glu254), lysine (Lys168, Lys179), threonine (Thr171, Thr172). According to the docking score of − 254.23, the ligand and receptor have an excellent binding affinity (Supplementary Table T4). These interactions imply that the aptamer functionalized nanoparticles can specifically bind to the EpCAM protein overexpressed in colorectal cancer tissue for the site-specific delivery of the drug [43, 44].

The aptamer was conjugated to drug-loaded nanoparticles via the EDC-NHS coupling reaction [45]. We confirmed successful aptamer conjugation using an agarose gel electrophoresis. As expected, the unconjugated aptamer migrated through the gel, whereas heavier Apt-2cNP (aptamer + 2c + nanoparticle) did not migrate and remained very close to the gel well. The 2cNP (2c + nanoparticle) system was undetectable since the formulation does not contain any DNA nucleotide sequence aptamer (Fig. 1A). We also confirmed conjugation using X-ray photoelectron spectroscopy, which clearly showed the expected peaks for nitrogen at 400.77 eV, 401.18 eV, and 401.26 eV for 2c, 2cNP, and Apt-2cNP, respectively. The peak height (11,955.56 CPS) and peak area (54,124.7 CPS.eV) for Apt-2cNP were higher than those for 2cNP (peak height 9966.02 CPS and peak area 45,046.93 CPS.eV), indicating the presence of a higher concentration of nitrogen originating from the presence of aptamers (Supplementary Figure S2).

### Evaluation of drug release profile for nanoparticles

We measured the in vitro drug release (Fig. 1E) and release kinetics in PBS (pH 7.4), PBS with 0.5% β-cyclodextrin (to enhance the solubility of the hydrophobic drug), and sodium acetate buffer of pH 5.5 to mimic the acidic pH in the TME, at several time points and analyzed the data using zero-order, first-order, Higuchi, Hixson–Crowell and Korsmeyer–Peppas equations (Supplementary Table T5). At 72 h, 37 ± 4.4%, 42 ± 5%, and 68 ± 4% of the drug were released in PBS, PBS with 0.5% β-cyclodextrin and sodium acetate buffer (pH 5.5), respectively. The release kinetics of the Apt-2cNP followed the Higuchi kinetic model in PBS and PBS + 0.5% β-cyclodextrin, with R^2^ of 0.963 and 0.951; and for sodium acetate buffer, it followed First order kinetics with regression coefficient (R^2^), 0.939.

### BA analogue 2c interacts more efficiently to topoisomerase-II than BA to inhibit the enzyme function

We noticed in our previous study that 2c intercalates into double-stranded DNA, causing DNA degradation after a 24-h incubation [46]. This degradation could be mediated by interacting with topoisomerases, which are enzymes that relieve tension on the DNA molecule during replication by making single-stranded (topoisomerase I) and double-stranded (topoisomerase II) breaks. We first explored the interaction of 2c with topoisomerases using the docking software AutoDock 4.2 and the Genetic-Lamarckian algorithm. The compound 2c contains more polar parts than its parent compound, BA, due to the attachment of a 1,4-disubstituted triazole ring, which makes it more likely to bind protein (Fig. 1F). Indeed, the docking studies show that 2c binds topoisomerases more strongly than BA, with a preference for topoisomerase-II over topoisomerase-I (Fig. 1G). The compound 2c binds at the active site of topoisomerase-II through hydrogen bonding and hydrophobic interactions with Arg690, Lys700, Pro701, Gly702, Lys 705, Tyr734, Glu808, and Ile833 (Fig. 1H, I). This could inhibit the enzyme function.

### Apt-2cNP is more cytotoxic than 2cNP to colorectal *cancer* cells and ensures greater cellular internalization

**Fig. 2:**
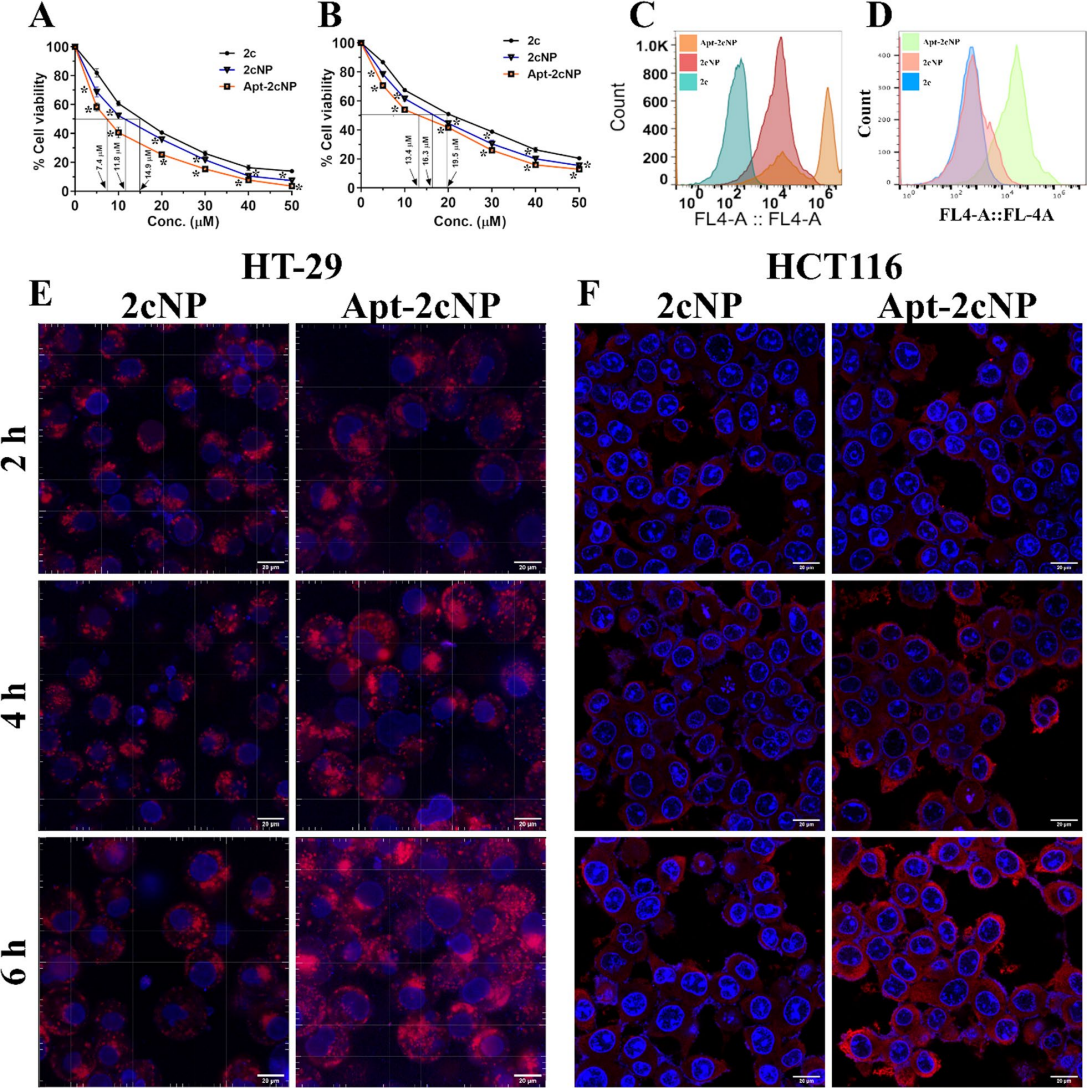
Apt-2cNP demonstrated enhanced cellular internalization and the highest cytotoxicity. Cytotoxicity of 2c, 2cNP, and Apt-2cNP was determined by MTT assay on (**A**) HT-29 and (**B**) HCT116 cells. Flow cytometric analysis of the intracellular uptake in (**C**) HT-29 cells for 2c (1st histogram, sea green), 2cNP (2nd histogram, reddish brown) and Apt-2cNP (3rd histogram, light orange) as well as in (**D**) HCT116 cells for 2c (1st histogram, Sky blue), 2cNP (2nd histogram, light red) and Apt-2cNP (3rd histogram, light green) at 6 h. Confocal microscopy of (**E**) HT-29 and (**F**) HCT116 cells treated with 2cNP and Apt-2cNP for 2 h, 4 h and 6 h (Blue = DAPI, Red = Cy5). Data shows mean ± SD (n = 3). *p < 0.05 was considered as the statistical level of significance

After formulating and characterizing Apt-2cNP, we evaluated the cytotoxicity of Apt-2cNP on two colorectal cancer cell lines, HT-29 and HCT-116, and the IC_50_ values obtained in them were 7.4 µM and 13.4 µM, respectively (Fig. 2A, B). We next exposed colorectal cancer cells HT-29 and HCT-116 to free drug, 2c, 2cNP, and Apt-2cNP treatment to quantify cellular uptake using flow cytometry (Fig. 2C, D), and we also performed confocal microscopy imaging for 2cNP and Apt-2cNP on HT-29 and HCT-116, respectively. We found a time-dependent increase in the cellular uptake for Cy5 loaded 2cNP and Apt-2cNP in HT-29 and HCT-116 cells, with the highest median fluorescence intensity at 6 h (maximum investigating period for the study) of Apt-2cNP treatment and an accumulation in the perinuclear region and little presence in the nuclear region, too (Fig. 2E, F).

### Apt-2cNP-induced apoptosis and mitochondrial depolarization in colorectal *cancer* cells

**Fig. 3:**
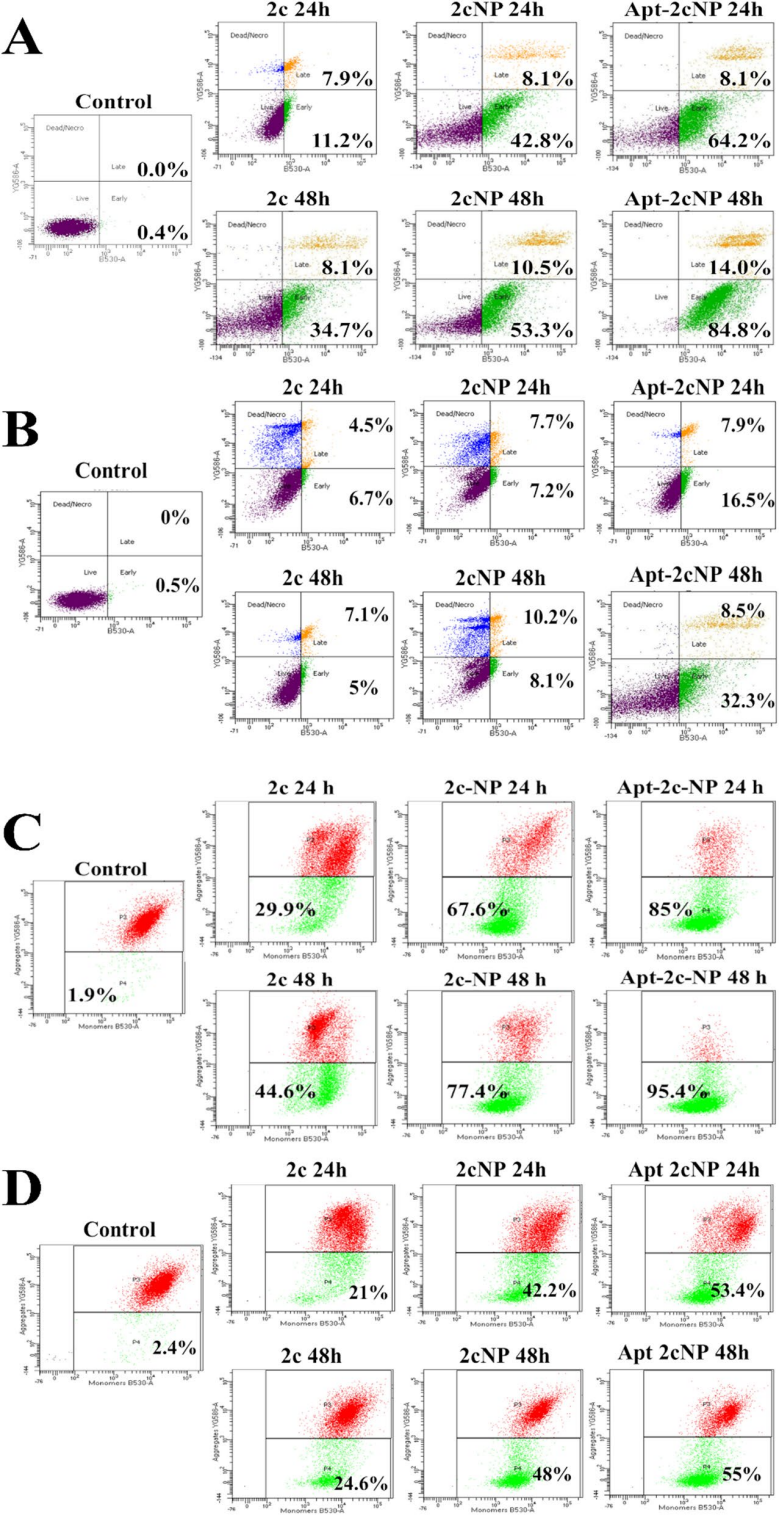
Apt-2cNP deciphered the highest efficiency to induce intrinsic apoptotic pathway. Representative images of apoptotic induction measured by flowcytometry after Annexin-V/PI dual staining on (**A**) HT-29 cells and (**B**) HCT116 cells. Representative images of mitochondrial membrane depolarization determined by flowcytometry after JC-1 staining on (**C**) HT-29 cells and (**D**) HCT116 cells after treatment with 2c, 2cNP, and Apt-2cNP for 24 h and 48 h

Initially, we measured the proportion of apoptotic HT-29 and HCT-116 cells after treatment with 2c, 2cNP, and Apt-2cNP at different time points using Annexin-V/PI dual staining (Fig. 3A). The percentage of apoptotic cells was 71.8 ± 2.4% (p < 0.05) and 98.33 ± 1.8% (p < 0.05) after 24 h and 48 h of treatment with Apt-2cNP, respectively, compared to 2cNP having 52.9 ± 1.88% (p < 0.05) and 64.8 ± 2.55% (p < 0.05) apoptotic cells, respectively for HT-29 cells. In HCT-116 cells, although a similar trend was observed, the apoptotic percentages were comparatively low (Fig. 3B). Since apoptosis is associated with mitochondrial depolarization, we analyzed mitochondrial membrane potential on HT-29 and HCT-116 cells (Fig. 3C) using JC-1 staining. As expected, we observed that Apt-2cNP treatment caused mitochondrial depolarization in 85.5 ± 2.64% (p < 0.05) and 96.1 ± 1.47% (p < 0.05) of cells at 24 h and 48 h, respectively, whereas 2cNP caused mitochondrial depolarization in 68.33 ± 1.53% (p < 0.05) and 77.13 ± 2.8% (p < 0.05) of HT-29 cells (Fig. 3C), respectively. A similar trend with lower values was observed in the case of HCT-116 cells that received 2c/ 2cNP /Apt-2cNP treatment for 24 and 48 h (Fig. 3D).

### Apt-2cNP inhibits cell cycle progression more than 2cNP

**Fig. 4:**
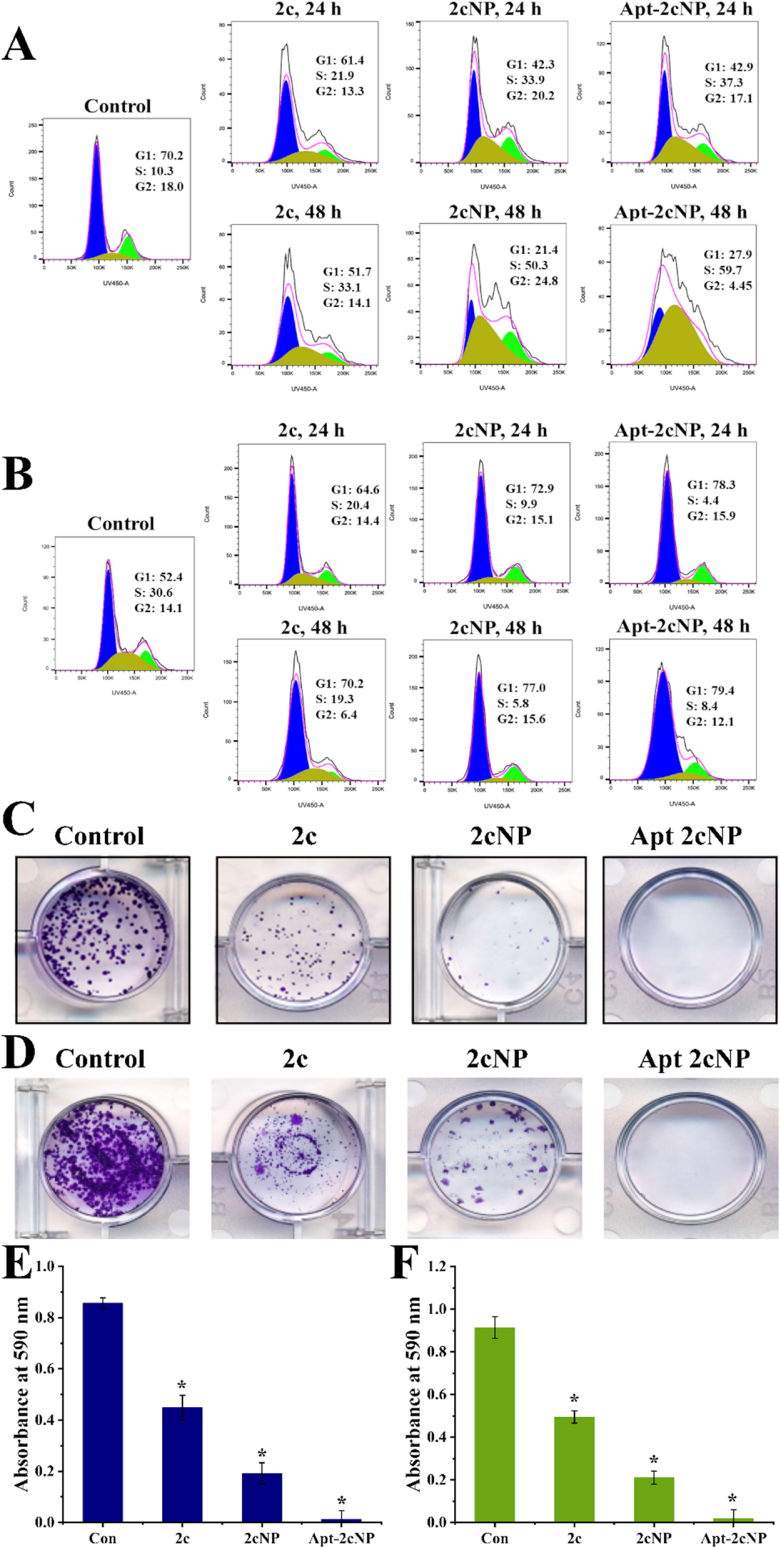
Apt-2cNP efficiently hault cell cycle progression and induced anti-tumorigenic effect. Cell cycle analysis was performed by flowcytometry on (**A**) HT-29 cells and (**B**) HCT116 cells after treatment with 2c, 2cNP, and Apt-2cNP for 24 h and 48 h. Representative images of clonogenic assay demonstrating inhibition of colony formation after treating (**C**) HT-29 cells and (**D**) HCT116 with 2c, 2cNP, and Apt-2cNP for 14 days. Quantitative representation of the potential of colony inhibition on (**E**) HT-29 and (**F**) HCT-116 cells. Error bars denote mean ± SD (n = 3), where * p < 0.05 is the statistical level of significance as determined by Student’s t-test

Different cytotoxic agents block the progression of the cell cycle at different phases. We found that 2cNP arrested the cell cycle of HT-29 cells in the S-phase, along with a little increase in the G2/M ratio. The population of S-phase arrest was observed to be 32.2 ± 1.7% (p < 0.05) and 50.8 ± 2.4% (p < 0.05) after 24 h and 48 h treatment with 2cNP**,** respectively, while the S-phase population in control cells was ~ 10%. For Apt-2cNP, an enhanced S-phase arrest was observed, with 36.9 ± 1.34% (p < 0.05) and 60.9 ± 1.3% (p < 0.05) of cells in S phase at 24 h and 48 h respectively (compared to 2cNP p < 0.05, Fig. 4A). In HCT-116 cells at 24 h, S-phase arrest (%) values were in the order of the treatment of 2c > 2cNP > Apt-2cNP **(**Fig. 4B), whereas, in G1phase, the value was maximum in Apt-2cNP followed by 2cNP. In G2 phase, the results were more or less similar in 2cNP and Apt-2cNP. At 48 h, there were increased values in G1 phase upon Apt-2cNP and 2cNP treatments. However, the value was marginally increased in Apt-2cNP compared to its value at 24 h. Similar trend was seen in the case of G2 pahse for 2cNP treatment. Interestingly S-phase value was decreased upon 2cNP treatment for 24 h, whereas the value was increased by twofold in the case of Apt-2cNP treatment at 48 h.

### Apt-2cNP efficiently controls cancer cell colony formation

In addition, in an HT-29 colony formation assay, the free drug (2c) produced a moderate prohibition in colony formation, whereas 2cNP more efficiently controlled colony formation ability (Fig. 4C), and the highest inhibition was observed after treatment with Apt-2cNP. For HCT-116 cells, the number of colonies was formed in a similar order; the number of colonies was quantified by the absorbance of dissolved crystal violet for the individual wells at 590 nm (Fig. 4D). Maximum inhibition was obtained upon Apt-2cNP treatment in both cell lines.

### Apt-2cNP induces autophagic cell death

**Fig. 5:**
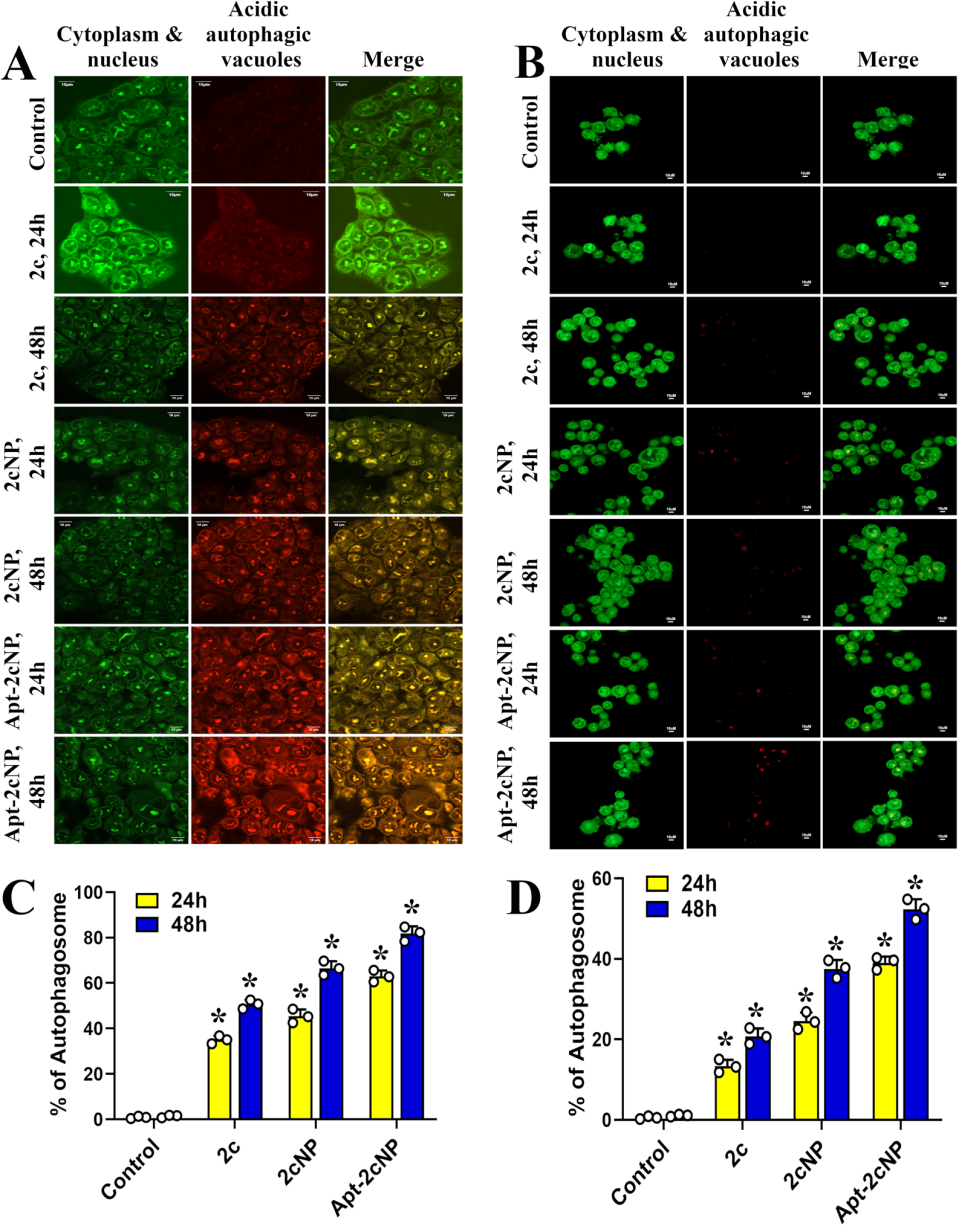
Apt-2cNP can induce autophagic programmed cell death. Autophagic cell death induction was monitored by acridine orange staining on (**A**) HT-29 and (**B**) HCT116 cells after treatment with 2c, 2cNP and Apt-2cNP for 24 h and 48 h as visualized under confocal laser microscope using filters for acridine orange (green, Excitation/ Emission at 500 nm/ 525 nm), with green depicting the cytoplasm and nucleus and (red, Excitation/ Emission at 460 nm/ 650 nm) orange/red depicting acidic compartments. Determination of autophagic flux formation by flow cytometry using fluorescent probe Cyto-ID Green staining after treatment with 2c, 2cNP, and Apt-2cNP for 24 h and 48 h. The bar diagram represents the percentage of autophagosomes in (**C**) HT-29 and (**D**) HCT116 cells. Error bars indicate mean ± SD (n = 3), where * p < 0.05 is the statistical level of significance as analyzed by Student’s t-test

We observed in our previous study that apoptosis was induced by 2c [7]. However, a variable findings were observed for HT-29 and HCT-116 cells. So, we investigated the effectiveness of Apt-2cNP to induce autophagic cell death, if any. We treated HT-29 and HCT-116 cells (Fig. 5A, B) with 2c, 2cNP, and Apt-2cNP for 24 h and 48 h and stained with acridine orange. Acridine orange emits green fluorescence when bound to the nucleus, but when it accumulates in acidic vacuoles, such as the lysosomes and autophagosomes indicative of autophagy, it emits red fluorescence [47]. Apt-2cNP induced more red fluorescence in treated cells than did 2cNP, with a peak at 48 h (Fig. 5A). To further confirm these acidic vacuoles associated with autophagy, we treated the colorectal cancer cells with 2c, 2cNP, and Apt-2cNP for 24 h and 48 h, followed by staining with fluorescent probe Cyto-ID Green, specifically accumulated at autophagosome and autolysosome compartments. By flow cytometric analysis, we found a time-dependent increase in autophagic flux formation compared to control cells, suggesting induction of autophagic cell death (Fig. 5C). However, Apt-2cNP-treated HT-29 cells had more autophagosomes (%) than that in HCT-116 cells (Fig. 5C, D).

### Hemolytic study indicates the formulations to be safe for intravenous use

A hemolytic study provides the comprehensive safety profile of a drug/formulation while in blood. The hemolytic activity for 2cNP and Apt-2cNP was low (< 5%) in comparison to free 2c at different concentration levels (Supplementary Figure S3), indicating the nanoformulations to be safe for intravenous administration within the tested dose range.

### Apt-2cNP activates pro-apoptotic genes and inhibits anti-apoptotic genes in CRC-TME

**Fig. 6:**
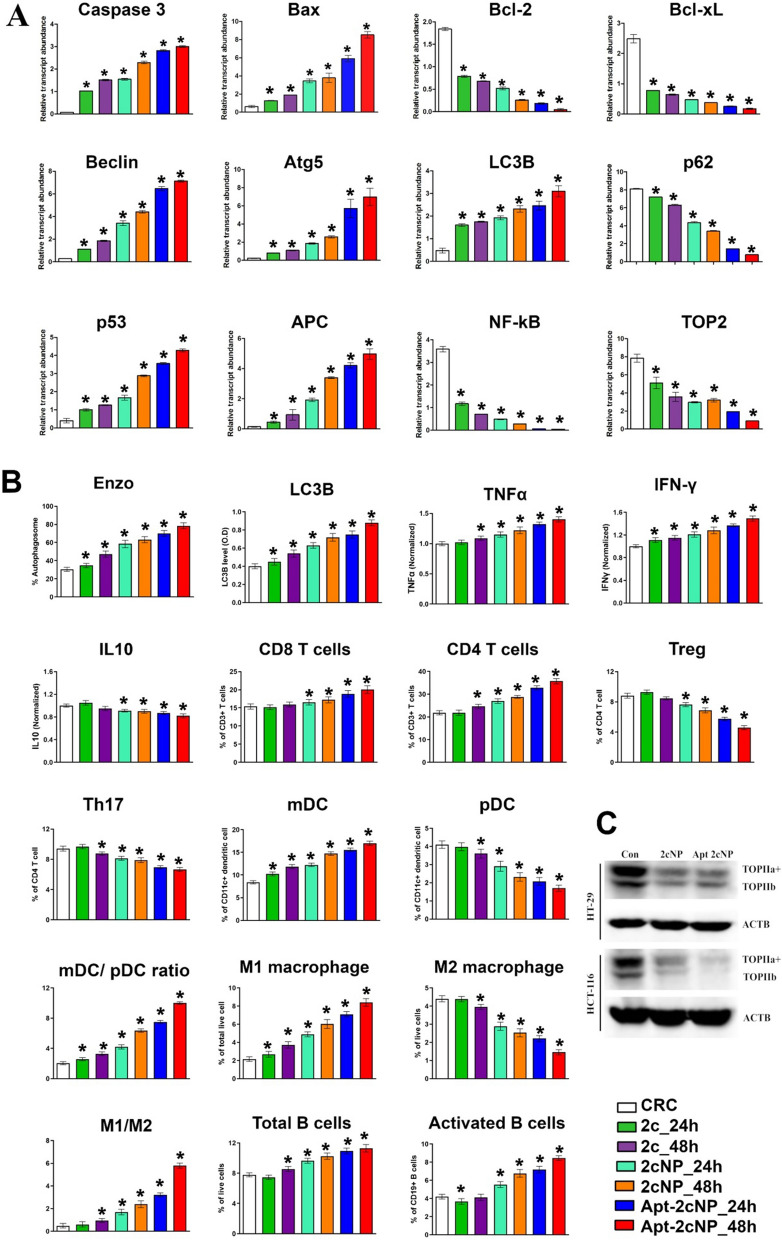
Anti-tumorigenic effect of nanotherapies in CRC tumor milieu. **A** qPCR analysis of apoptotic genes (Caspase 3, Bax, Bcl-2, and Bcl-XL), autophagy-related genes (Beclin, Atg5, LC3B, and p62), and selected genes related to the mechanism of action of BA (p53, APC, NF-kB and Topoisomerase-II, TOP2) with RNA isolated from tumor tissues of CRC mice model. **B** Bar diagrammatic representation of in vivo autophagosome % and LC3B expression level. Measurement of cytokine profile, TNFα, IFNγ, IL-10 level, and quantification of different immune cell populations (T cells, dendritic cells, macrophages, and B cells) in the TME by flowcytometry with the cells isolated from tumor tissue after treatment with 2c, 2cNP, Apt-2cNP at different time points (24 h, 48 h) and without treated CRC mice considered as the control group. **C** Western blot of in vitro expression of Top-IIa and Top-IIb proteins isolated from HT-29 and HCT-116 cells after 48 h treatment with 2cNP and Apt-2cNP. Error bars indicate mean ± SD (n = 3), where *p < 0.05 is the statistical level of significance as analyzed by Student’s t-test

We monitored the overall effect of Apt-2cNP on two different types of cell populations in CRC-TME. To test in vivo apoptosis induction, we treated the CRC mouse model with 2c, 2cNP, or Apt-2cNP and found a time-dependent increase in the RNA expression of pro-apoptotic genes (*caspase 3* and *Bax*) and a decrease in anti-apoptotic genes (*Bcl-2* and *Bcl-XL*) after 24 h and 48 h treatment (Fig. 6A). In all the cases, Apt-2cNP had a more profound effect than 2c and 2cNP. Thus, aptamer conjugation to specifically target colorectal cancer cells improved both the in vitro and in vivo apoptotic efficacy in the animal colorectal cancer model (Fig. 6A).

Furthermore, to test for autophagy in vivo, we treated a CRC mouse model with 2c, 2cNP, and Apt-2cNP and found a time-dependent increase in the RNA expression of autophagy-related genes (*Atg5*, *LC3B*, *Beclin*) and a decrease in p62 expression 24 h, or 48 h after treatment. Additionally, we stained a single-cell suspension of epithelial cells isolated from CRC mice tumor tissue with a dye that detects autophagic vacuoles (Cyto-ID) (Fig. 6B), suggesting a significant increase of the stained cells in Apt-2cNP (P < 0.05) than the 2cNP and the control. Concordantly, we also found a time-dependent increase in LC3B expression (p < 0.05 after 48 h treatment), with Apt-2cNP having a more predominant effect than 2cNP (p < 0.05 after 48 h treatment).

### Apt-2cNP modulates immune response in TME

CRC heterogeneous tumor microenvironment includes not only neoplastic cells but also various immune cell types that play a crucial role in cancer initiation, progression, and metastasis development. Accumulating evidences suggest that chemotherapeutic drugs can also modulate immune cells and induce anti-tumorigenic immune response CRC microenvironment [35]. Therefore, we investigated the functions of different categories of immune cells in the TME of CRC mice after treatment with 2c, 2cNP, and Apt-2cNP for 24 h and 48 h. We evaluated the cytokine profiles of cells isolated from CRC tumor tissue and observed a time-dependent increase in the expression of the pro-inflammatory cytokines TNFα and IFNγ for all different treatment groups, whereas downregulation of anti-inflammatory cytokine IL10 (Fig. 6B). Next, we investigated the effector immune cells in CRC tissue, including CD8 T cells, dendritic cells, B cells, and the cassical macrophage population. The percentages of CD4 and CD8 T cells were found to significantly increase after treatment with 2cNP and Apt-2cNP in a time-dependent manner (Fig. 6B, Supplementary Figure S4). However, those populations did not change after free drug treatment. The antigen-presenting cells (APCs) in cancer immune microenvironments, especially myeloid DC (mDC) and M1 macrophage population level, enhanced after treatment with different free drugs and nanotherapies (Fig. 6B, Supplementary Figure S5, S6). Additionally, we studied another important immune cell population, B cells, which have a significant role in anti-tumor immunity through antibody-dependent toxicity and as APC for T-lymphocytes. We noticed a significant increase in CD19 + total B cell population after treatment with nanoencapsulated 2c (Fig. 6B, Supplementary Figure S6). Furthermore, we studied the MHC-class II positive population percentage to identify the activated B cells. A significant increase in the CD19 + MHC-II + population was observed in the case of 2cNP and Apt-2cNP.

Apart from effector immune cells, we also focused on the suppressive population of immune cells in TME, including plasmacytoid DC (pDC), M2 macrophage population, and regulatory T cells (Treg). A time-dependent decrease in the pDC, M2, and Treg cell populations (Fig. 6B) was observed after 2cNP and Apt-2cNP treatment, which supports an anti-tumorigenic immune microenvironment.

### BA analogue 2c notably inhibits topoisomerase-II

Using CRC mouse model, we found that treatment with 2c, 2cNP, or Apt-2cNP (24 h, and 48 h after treatment) caused a time-dependent decrease in the RNA level that correlates well with in vitro protein expression of topoisomerase-II in colorectal cancer cell lysate (Fig. 6C). Thus, 2c notably inhibits topoisomerase-II to effectively block DNA replication during the S phase of the cell cycle.

### Apt-2cNP accumulates in the cancerous regions of the colon and reduces tumor growth in vivo

**Fig. 7:**
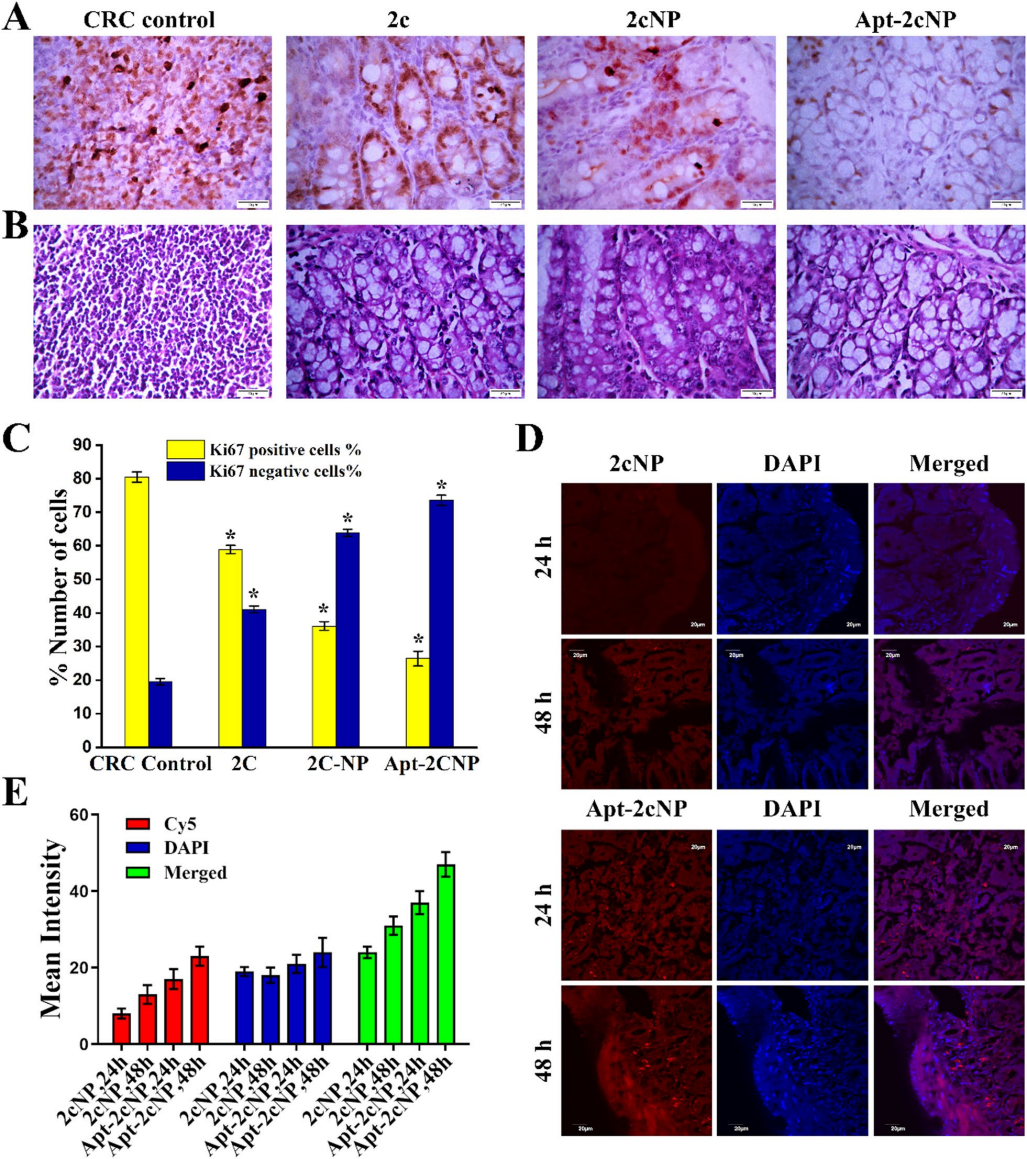
Determination of in vivo healing efficacy by (**A**) H&E staining and (**B**) Ki67 immunohistochemistry for colon isolated from experimental CRC mice and from CRC mice after treatment with 2c, 2cNP, Apt-2cNP (Magnification of each panel 40 ×). **C** Quantification of Ki67 immunohistochemistry assay with bar diagrammatic representation for CRC control and different treated groups. In vivo tumor tissue distribution of 2cNP and Apt-2cNP by (**D**) confocal microscopy measured in the colons of CRC mice after intravenous administration of Cy5-loaded 2cNP and Apt-2cNP. **E** The relative intensity of Cy5 (representing 2cNP and Apt-2cNP), DAPI, and Merged images for each panel were measured through Image J software. Data plotted as mean ± SD of three individual measurements, where *p < 0.05 is the statistical level of significance as determined by Student’s t-test

After confirming an in vivo functional activity, we next investigated whether Apt-2cNP had a therapeutic efficacy. To test for therapeutic healing, we isolated colons from CRC mice with and without treatment. Histological analysis of colonic tissue by H&E staining revealed the improved tissue condition after treatment with 2c, 2cNP, and Apt-2cNP in comparison to the untreated CRC control group. The highest healing efficacy was observed in the case of Apt-2cNP treatment (Fig. 7A). Treating CRC mice with Apt-2cNP reduced carcinoma spreading and cancerous growth in the colons by 67.5% (p < 0.05) (Fig. 7B) as the percentage of ki67 positive cells in CRC was 80.45 ± 1.5 and that was found to reduce 26.44 ± 2.2 in the case of the Apt-2cNP treated mice. Furthermore, in contrast to the untreated CRC control, the treated groups (2c, 2cNP, and Apt-2cNP) had fewer Ki67-positive cells in their colons 58.88 ± 1.2% (p < 0.05), 36.15 ± 1.3% (p < 0.05), and 26.44 ± 2.2% (p < 0.05) respectively, indicating that Apt-2cNP showed the maximum inhibitory effect against cancer cell proliferation (Fig. 7B, C). These findings confirm that the tumor cells were suppressed following treatment, with the highest efficacy for Apt-2cNP. We next conjugated Cy5 to Apt-2cNP and investigated the colons of treated CRC mice, indicating that Apt-2cNP accumulated maximum in cancerous regions of the colon after 48 h of Apt-2cNP treatment (Fig. 7D, E), that well correlates with the highest therapeutic effect of Apt-2cNP to reduce tumor burden in CRC murine model.

### Pharmacokinetic and biodistribution profiles of the experimental formulations

**Fig. 8:**
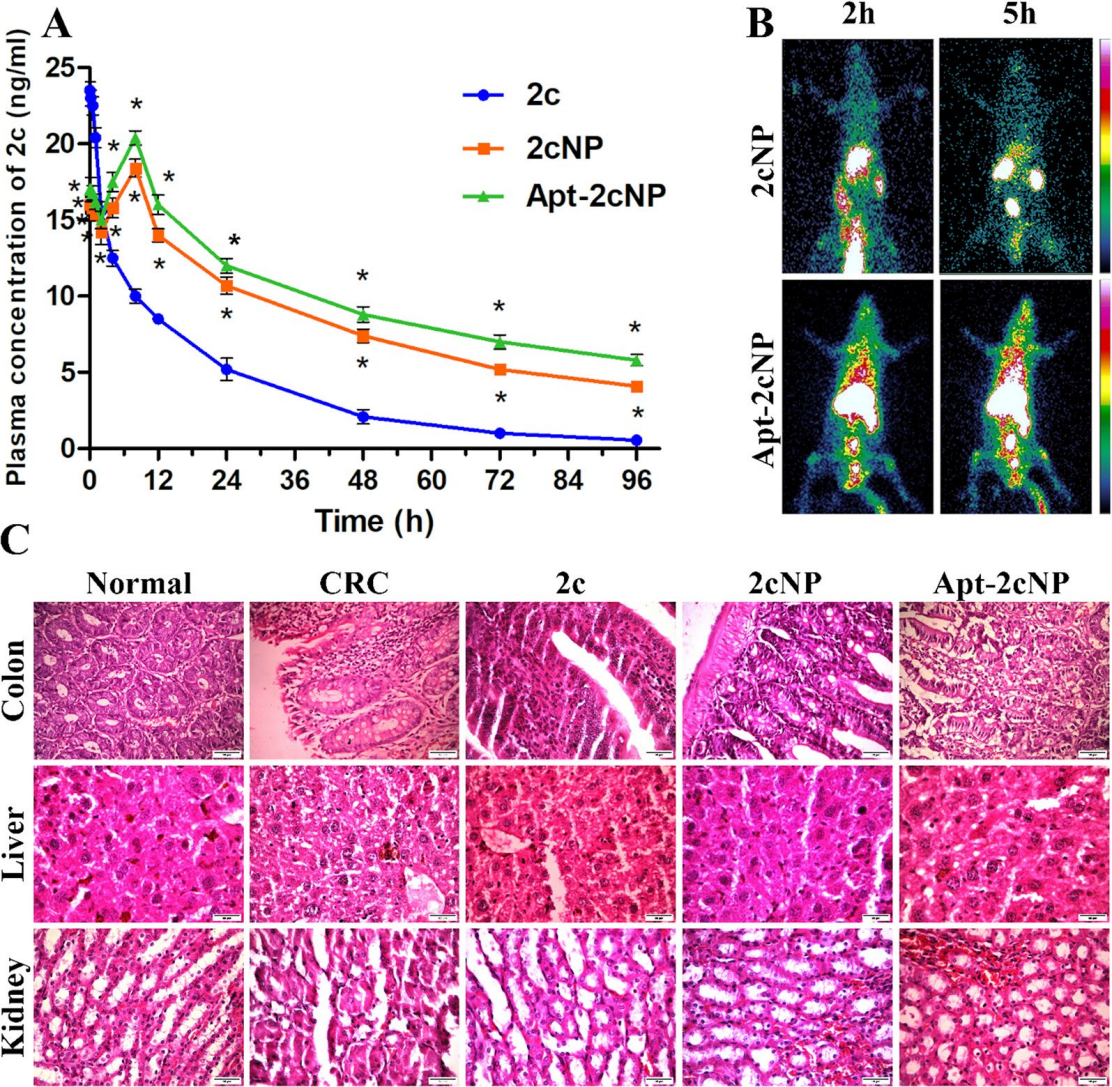
Systemic availability and in vivo healing efficacy of free drug and nanoparticles evaluated by pharmacokinetic profiling.** A** Plasma concentration vs time plot representing concentration of 2c in mouse plasma at different time points after administering 2c/ 2cNP/ Apt-2cNP. Tumor tissue accumulation of 2cNP and Apt-2cNP in CRC rat model by (**B**) Gamma scintigraphy live imaging of rats after the injection of ^99m^Tc-labeled 2cNP and Apt-2cNP through the tail vein at 2 h and at 5 h. The scale on the right represents relative color intensity. Evaluation of in vivo toxicity of Apt-2cNP in major organs of mice measured by (**C**) Histopathological morphology of colon, liver, and kidneys isolated from normal animals, experimental CRC-bearing animals, and from CRC-bearing animals after treatment with 2c, 2cNP and Apt-2cNP (magnification of each panel 40 ×). Error bars indicate mean ± SD (n = 3), where *p < 0.05 is the statistical level of significance as analyzed by Student’s t-test

Additionally, we cataloged the pharmacokinetics and biodistribution of Apt-2cNP. Systemic availabilities of 2c after intravenous administration of 2c, 2cNP, and Apt-2cNP were observed through plasma concentration measurement of 2c at the different experimental time points (Fig. 8A). In the case of the free drug 2c, the peak plasma concentration was found just after the injection (1 min), while in the cases of 2cNP and Apt-2cNP, it was achieved at around 8 h of injection. The AUC values were increased by 2.18 times and 2.58 times for 2cNP and Apt-2cNP, respectively, compared to free drug 2c. Significant changes in t_1/2_ and AUMC values have been observed in the case of 2cNP and Apt-2cNP, compared to 2c (Supplementary Table T6). In the case of 2c, the half-life was found to be 6 ± 0.5 h, while for 2cNP and Apt-2cNP, those values were 34.0 ± 1.5 h and 36.0 ± 1.0 h, respectively. The mean value of AUMC was increased by 3.46 times and 4.36 times in the cases of 2cNP and Apt-2cNP, respectively, compared to the free drug 2c. Mean retention time (MRT) in plasma was also enhanced significantly for 2cNP and Apt-2cNP, suggesting that retention of 2c was enhanced upon nanoencapsulation.

To clearly assess the distribution of Apt-2cNP throughout the body, we injected ^99m^Tc-radiolabeled nanoparticles (^99m^Tc-Apt-2cNP/^99m^Tc-2cNP) into comparatively larger CRC-bearing animal model, Sprague–Dawley rats for 1 h, 2 h, and 5 h, and observed the distribution using a gamma scintigraphic camera. The majority of the injected nanoparticles accumulated in the liver, with a significant accumulation in the colon, both increasing with the progression of treatment time (Fig. 8B). The liver is the largest and most vital metabolic organ that eliminates most foreign and indigenous chemicals [48]. A recent report suggests that over 99% of nondegradable and hard nanomaterials, such as quantum dots, silica nanoparticles, gold nanoparticles, etc., undergo fast hepatic clearance [49]. Soft nanoparticles, such as polymeric nanoparticles, liposomes, etc., are also eliminated by the liver to a notable extent. New therapeutic design approaches, such as targeted monotherapy, can invade hepatic tumors and internalize them into cancer cells through cellular endocytosis [50]. After the endosomal escape while in cytoplasm, they release drugs in the cells for specific functions [51]. Thus, passive and active drug targetings to specific liver cells or tumors can avoid or delay the hepatic elimination of nanostructures [52].

At 5 h post-injection, a large amount of Apt-2cNP accumulated in the distal part of the colon, indicating the presence in the cancerous region. After isolating and staining different parts of the colon, we confirmed that the regions where ^99m^Tc-Apt-2cNP accumulated more in cancerous than the surrounding parts of the tissue. The major elimination of the radiolabeled nanoparticles occurred through urinary excretion. The radioactivity present in different organs or body fluids (biodistribution) was calculated as the percent of injected dose and is shown in Supplementary Table T7. CRC in these mice was induced by a carcinogen, DMH, which can also affect the liver and kidneys [53, 54]. After sectioning and staining, the colons of control mice without CRC showed distinct, healthy layers of cells, whereas CRC mice had abundant cancerous regions (adenomatous polyposis coli) throughout the epithelial layers of their colons. In some portions of the colon, the carcinoma regions spread up to the submucosal layer through the tunica mucosa and became considerably larger in size. As expected, cells inside the polyps were loosely arranged and had large nuclei. Concordantly, we found histological changes in the liver and kidney of CRC mice, with the liver having minor changes in hepatocyte architecture, with a few pyknotic and karyorrhexis nuclei, and the kidney having slightly atrophic glomeruli (Fig. 8C). However, after treatment with Apt-2cNP, the histology of these tissues was improved. We found that the restoration of classic hepatic lobules and healthy blood sinusoids in the liver and normal renal tubular structure and glomeruli in the kidneys, indicating not only healing efficacy but also non-toxic to off-target tissues.

## Discussion

The distribution of drugs in tumors and their surrounding normal tissues is one of the major hurdles in chemotherapy for non-specific drug delivery and dose-related toxicities. It hinders the clinical success of many drug candidates. One of the promising approaches for delivering drugs to the targeted cancer tissues is the neoplastic cell-specific receptor-targeted delivery system loaded with chemotherapeutic agents. Further, target-specific ligand-conjugated polymeric nanoparticulated delivery systems hold significant promise for delivering hydrophobic drugs in the effective drug concentration, specifically in the tumor area [55, 56]. Here, 71 bp DNA aptamer was surface-conjugated with the 2c-loaded PLGA nanoparticle. The compound 2c is a promising anticancer compound [46]. The accuracy of the aptamer binding with the overexpressed EpCAM receptors in the colorectal cancer cells was initially verified using molecular docking.

We selected an aptamer as a targeting ligand due to its small size, high tissue penetration capability, and very low immunogenicity compared to antibodies, peptides, and other targeting molecules. The receptor-specific binding of aptamer-conjugated nanoparticles is attributed to their faster attachment with the cell membrane [17]. Aptamer-conjugation with the 2cNP was investigated by gel electrophoresis. The result was further substantiated by X-ray photoelectron spectroscopy (XPS) study. XPS is an effective method for examining the surface of a particular sample and detecting the elemental composition of the surface. In the present investigation, the free-drug 2c, which possesses a nitrogen-rich triazole ring, showcases a high nitrogen percentage of 5.25%. However, the 2cNP and Apt-2cNP displayed nitrogen percentages of 1.44% and 1.8%, respectively. The encapsulation of the free drug (2c) into the PLGA NP reduced the availability of drug (2c) molecules on the surface. Notably, the atomic percentage of nitrogen in the 2cNP was less compared to Apt-2cNP. The conjugation of nitrogen-rich DNA aptamer increased the nitrogen percentage on the surface of Apt-2cNP.

The morphology of the designed nanoparticles examined by FESEM, AFM, and cryo-TEM techniques represents a spherical shape, smooth surface, and aggregation-free stable particles having an average size range of 180–188 nm. Many particles even had sizes of 100 nm and below. Nearly 8% drug loading and around 87% encapsulation efficiency suggest good drug encapsulation and little material loss during manufacturing, even at a small scale. Negative zeta potential was found to be more negative in value due to aptamer incorporation, as the aptamer had a negative charge. Negative zeta potential values suggest that the particles should be stored in a free-dried form and should be suspended before use.

An in-vitro drug release study usually explains how the encapsulated drug would be released from the formulation while in the blood [57]. The present investigation showed that from all the formulations, the drug release was fast in the first 24 h, and eventually, the drug release pattern was slow and sustained. The presence of drug particles near the surface of the nanoparticles might diffuse faster, causing initial fast drug release. The findings suggest that the nanoparticles would provide the initial drug blood level by the early faster drug release, followed by a sustained drug action.

The drug release was investigated in phosphate buffer saline (PBS, 7.4) with or without 0.5% w/v β-CD (β-cyclodextrin). β-CD was added to prevent drug saturation in the release media. The trend of drug release was more or less similar. The drug release was higher in PBS with β-cyclodextrin. However, maximum and faster drug release was noted in sodium acetate buffer (pH 5.5), which suggests that the drug would release more in the tumor. The pH within a tumor is always acidic, varied between pH 5.5 and pH 6.5 [58].

Further, the drug release data were tested on various release kinetic models, such as zero-order, first-order, Higuchi kinetic, Korsmeyer-Peppas, and Hixon-Crowell. Data suggests that the drug release primarily followed the Higuchi kinetics [59].

Drug release from Apt-2cNP followed the Higuchi kinetic model. It suggests that drug molecules diffused from the insoluble matrix at a rate proportional to the square root of time and drug release following Fickian diffusion [60] with a sustained drug release pattern. Sustained drug release would help maintain drug concentration in the tumor cells for a much longer time, prolonging neoplastic cellular cytotoxicity and causing faster apoptosis and cancer cell death.

Using AutoDock 4.2 and the genetic-Lamarckian algorithm, we investigated the compound 2c and topoisomerase interaction and confirmed a preferentially strong binding of the 1,4-disubstituted triazole ring of 2c with topoisomerase-II over topoisomerase-I. This molecular interaction of 2c with topoisomerase-II could inhibit the function of the enzyme and block cell division by inhibiting DNA synthesis in colorectal cancer.

We have found that the aptamer-conjugated nanoparticles (i.e., Apt-2cNP) controlled the progress of colorectal cancer and had the maximum cytotoxic effect on HT-29 cells. The confocal microscopy images and the flow cytometry analysis revealed a consistent accumulation of Apt-2cNP with time in HT-29. Apt-2cNP was distributed throughout the cells, including the periphery of the nucleus, reflecting the nanoparticles’ ability to release drug content in the perinuclear region. In HCT-116 cells, Apt-2cNP behaved similarly but with lesser efficacy, as supported by MTT assay and FACs data.

Annexin V/PI and JC1 stainings demonstrated the highest percentage of apoptotic cells upon Apt-2cNP treatment. Targeting the EpCAM receptor with the aptamer ensured efficient drug delivery specifically to colorectal cancer, followed by good cellular internalization. Acridine orange staining revealed the ability of Apt-2cNP to generate acidic vesicular organelles (AVO) more efficiently over 2cNP. Acridine orange is a hydrophobic dye that emits green fluorescence in neutral pH and becomes protonated in acidic conditions, emitting red fluorescence [61]. The fluorescent probe Cyto-ID staining outcomes fairly correlated with that of acridine orange staining and confirmed Apt-2cNP mediated stimulation of autophagic cell death. Apt-2cNP caused greater total apoptosis and mitochondrial membrane depolarization in HT-29 cells along with induction of autophagic cell death than in HCT-116 cells. This could be because of the higher Apt-2cNP cellular uptake in HT-29 cells than in HCT-116 cells.

We previously observed that 2c can intercalate into double-stranded DNA, causing nuclear degradation and ROS generation and inhibiting cellular proliferation [61]. This damage could be mediated by interactions with TOPI and II, which interact with the pre-replicative complex during the M phase, early G1, and G1/S phase to monitor the origin of replication [62]. The drug molecules act as topoisomerase-II poisons, block replication or the S-phase of the cell cycle, and have several toxic effects [19]. BA is an inhibitor of topoisomerase-II [63], which agrees with our finding that 2c also inhibits topoisomerase-II activity and, blocks the cell cycle at S-phase, and results in apoptosis induction in colorectal cancer cells.

Autophagic cell death can be stimulated by different types of cellular stresses, including DNA damage, protein aggregates, ROS generation, damaged organelles, and so on. Autophagy can play a dual role under different conditions. Sometimes, it promotes cell survival by eliminating damaged organelles and proteins. However, autophagy induction beyond a threshold limit causes cell death. The binding of 2c with double standard DNA and the possible damage mediated by topoisomerase II enzyme poisoning highly correlate with S-phase blockage and simultaneous induction of apoptotic and autophagic cell death.

Accumulating evidences suggest that autophagy has a pivotal and putative inverse relationship in cell-cycle progression in neoplasia [64]. Autophagy induction has been inversely correlated extensively with cell cycle arrest [64]. In basal autophagy, the cells undergo many structural rearrangements in the G1, S, and G2/M phases of the cell cycle, and inhibition of autophagy prevents undesired organelle/chromosome loss [64]. Autophagy, a key player in our study, suppresses tumors in the early stages but promotes cancer later on, leading to resistance to treatments. During early carcinogenesis, autophagy plays a crucial role in inhibiting neoplastic cell survival, metabolism, and energy production in response to carcinogenic insults. This insight into the role of autophagy in cancer growth provides a new avenue for potential treatment strategies.

In human cancer cells, the components of the cell cycle machinery alter recurrently. The cell cycle arrest (mostly at G1/S or G2/M boundaries) fails, causing uncontrolled neoplastic cell proliferation. In the present study, the S-phase arrest was increased most with Apt-2cNP application, indicating more extensive DNA synthesis inhibition and cell cycle arrest. Apt-2cNP-mediated efficient autophagy induction and increased S-phase arrest of the cell cycle support the inverse relationship between autophagy and cell cycle arrest. This promising result underscores the potential of Apt-2cNP as a potent anticancer agent. The findings are also well-implicated to provide a relationship between autophagy and cancer.

After the completion of the in vitro studies, we evaluated hemocompatibility, which is crucial for the effective in vivo application of blood-contacting biomaterials [36]. The US-FDA has recommended biodegradable PLGA as safe for human intravenous administration. Hemocompatibility in the present investigation suggests that Apt-2cNP/2cNP is equally safe for intravenous injection for their modest hemolytic activity.

Cancer cell invasion, called metastasis, is the primary cause of cancer-related death. The intracellular mechanistic modulations in malignancy influence neoplastic cell migration and invasion. Like many other natural compounds such as epigallocatechin-3-gallate (EGCG), resveratrol, apigenin, and genistein, BA effectively reduces cancer cell migration and invasion. Reports suggest that BA inhibited gastric cancer cell migration and invasion by impairing epithelial‐mesenchymal transition signaling [56, 65] and targeting the NF-κB/VASP signaling pathway [56, 65]. BA analogue 2c is more active than BA, and its formulation, Apt-2cNP, is remarkably efficacious in the current study. Hence, Apt-2cNP may also be a potential inhibitor of metastasis progression of colorectal cancer.

To further investigate the in vivo cell death mechanism, we analyzed the mRNA expression of different pro-apoptotic and anti-apoptotic genes after treatment with 2c, 2cNP, and Apt-2cNP in the CRC murine model. The significant upregulation of the pro-apoptotic genes and downregulation of anti-apoptotic genes after Apt-2cNP treatment have supported the 2c-mediated induction of the intrinsic apoptosis pathway as a mitochondrial-initiated event. Interestingly, a definitive increase in autophagy induction was also observed in the tumor microenvironment as a simultaneous event. The gene *p53* has several functions in apoptosis, cell cycle, and senescence of cells. Besides, its role in the activation of several genes involved in apoptosis (e.g., *Bax, Bad, Bak, Noxa, Puma*) enhances the secretion of cytochrome c from mitochondria into the cytosol and triggers the intrinsic pathway of apoptosis [66]. We also examined the expression of *APC,* as the gene reported to inactivate during the progression of colorectal cancer [67]. Another known mediator of cell growth and the inflammatory pathway is NF-κB [68]. Treatment of 2cNP/Apt-2cNP in CRC murine model downregulated *NF-κB* mRNA expression, suggesting the downregulation of NF-κB pathway after the treatment regimen.

Apt-2cNP reduced topoisomerase II activity (TOP2A and TOP2B), depolarized mitochondrial membrane potential, increased DNA fragmentation, and intrinsic caspase pathway, demonstrating increased expression of pro-apoptotic genes (*caspase 3* and *Bax*), and decreased anti-apoptotic genes (*Bcl-2* and *Bcl-XL*), and ultimately led to apoptosis. Apt-2cNP also showed a time-dependent increase in autophagy-related gene expression (*Atg5*, *LC3B*, *Beclin*), a decrease in p62 expression, and induced cell cycle arrest. Thus, in the present in vivo study, apoptosis and autophagy both suppressed tumor growth. Apoptosis inhibits cell survival, facilitating apoptotic signal activation, inhibiting anti-apoptotic signaling protein levels, and modulating biochemical cellular activities, thus inducing cell death. In contrast, autophagy arrested the cell cycle and manipulated autophagy genes to reduce tumor growth. Reports suggest that autophagy is inversely correlated with cell-cycle progression in neoplasia [64].

Chemotherapy has been considered as an immunosuppressive process for a long time. Effector immune cells and neoplastic cell populations in tumor microenvironment are also susceptible to cytotoxicity induced by chemotherapeutic treatment regimens. However, accumulating evidence revealed that many potent chemotherapies, including anthracyclines, taxanes, and topoisomerase inhibitors, can boost host immune response and antiproliferative activity [69]. In most cases, these drug molecules induce apoptotic bodies from dying cancer cells and are represented by antigen-presenting cells to T-lymphocytes. This phenomenon is named immunogenic cell death [35]. On the other hand, the direct effect of chemotherapeutic drugs on immune cells, such as dendritic cells, has also been reported [70, 71]. However, some chemotherapeutic drugs exert a sudden depletion of lymphocytes immediately after treatment, showing immunosuppressive effects in TME. Several recent reports reflected that a low dose treatment of cyclophosphamide (CTX) for a prolonged period can efficiently stimulate macrophages, dendritic cells, and NK cells in mice tumor models [72, 73].

Apart from studying proliferative activity, here we investigated the anti-tumorigenic effect of the free drug and its nanotherapies in the CRC murine tumor microenvironment. Despite nonsignificant changes in lymphocytes after free drug 2c treatment, the anti-tumor immunity of nanoencapsulated 2c and targeted nanotherapy was significantly enhanced at the CRC tumor site. The free drug may cause an initial lymphodepletion due to the cytotoxic effect; however, the sustained release of low drug concentration from nanoparticles can efficiently modulate the immune cell populations [69]. Apt-2cNP treatment efficiently reduced the Treg cell population while inducing the highest proliferation of CD8 T cells. In addition, 2cNP and Apt-2cNP augmented M1 macrophages, and the mDC population concurrently increased another important antigen, presenting activated B cells. These findings indicate that 2c nanoparticles can potentially modulate the effector immune cells with upregulation of pro-inflammatory cytokines, TNFα and IFNγ whereas downregulated anti-inflammatory cytokine, IL-10, along with Treg, Th17, pDC cell populations responsible for immunosuppressive CRC tumor milieu [74]. However, further investigation is warranted for a detailed study of immune microenvironment and mechanism for immune modulatory function [75-78].

The kinetic distribution of hydrophobic drug molecules encapsulated in polymeric nanoparticles with targeting moiety can effectively maintain in vivo plasma drug concentration [55]. To ensure its clinical application, the pharmacokinetic response of the drug is evaluated. Pharmacokinetic data of 2cNP and Apt-2cNP showed nanoparticles predominantly sustained the drug release in blood. However, aptamer conjugation on the nanoparticle surface (Apt-2cNP) further delayed the drug release pattern and other pharmacokinetic parameters related to sustained drug release. It suggests that aptamer has a role in the delayed drug release. Aptamers might produce an aqueous microenvironment surrounding the nanoparticles [75, 79], hindering the hydrophobic drug release from Apt-2cNP and altering the pharmacokinetic parameters. Confocal microscopy images reveal time-dependent accumulation of Apt-2cNP in CRC tissue of mice [7]. The nanoparticles were well-deposited in colonic crypts, even in the terminal regions at 5 h post-injection. Some regions of colonic epithelia were full of bright red spots indicative of receptors, particularly EpCAM-rich regions in cancerous colons. Biodistribution study and gamma scintigraphic imaging also revealed a good accumulation of aptamer-conjugated nanoparticles in the colonic region of CRC animal models. Both the distal and transverse parts of the colon were visible in gamma scintigraphic images of rats, suggesting the presence of a tumor region therein.

Microscopic observation of colon samples isolated from normal mice showed uniform distribution of the epithelial layer, clearly segmented with healthy cells of the mucosal and submucosal layer. Treatment with DMH (20 mg/kg once a week for 12 weeks) generated several polyps throughout the colon, most of which were microscopic and not visible from the outside. The presence of adenomatous polyps discontinued the epithelial homogeneity and disturbed normal homeostasis of the colon. Many colonic regions had excessive mucosa and were morphologically different cells. The cells inside the polyps were morphologically distinct from other epithelial cells (e.g., goblet cells) and had different size distributions and orientations [80, 81]. After the Apt-2cNP treatment, we found a significant decrease in the number of polyps and restoration of the epithelial layer. DMH treatment produced significant changes in the liver and kidneys of mice. The glandular structure of liver tissue became thinner in some liver regions. The glomerular structure and tubular arrangements were distorted to some extent. Interestingly, after treatment with Apt-2cNP, the histological condition of the liver and kidney was improved towards the normal stage, along with the improvement of colonic histopathology.

Targeted nanotherapy showcases several advantages over conventional therapy. They include precision targeting of specific cells or tissues by functionalizing nanoparticles with ligands or antibodies that recognize specific molecular markers to deliver drugs directly to the affected cells while sparing healthy ones. They enhance drug efficacy by enhancing their solubility, stability, and bioavailability and ensure optimal drug concentration at the site of action to maximize therapeutic effects and minimize off-target effects due to site-specific delivery, thereby reducing drug toxicity. Nanoparticles improve the pharmacokinetics profile of drugs by prolonging blood residence time, leading to sustained drug release and reducing dosing frequency.

Off note, targeted nanotherapeutic studies have some limitations, such as design and evaluation complexity, scaling up manufacturing, cost and regulatory hurdles, need for comprehensive safety and efficacy issues in humans, high development cost compared to conventional formulations, intellectual property issues, market acceptance, and reimbursement are some of the limitations of this present study.

However, here we have successfully developed and optimized efficacious Apt-conjugated 2c-loaded PLGA nanoparticles with high-encapsulation efficiency that can be administered intravenously and can release drug for an extended period, producing immunomodulation in the tumor microenvironment and killing cancer cells by apoptosis and autophagy. PLGA nanoparticles are successful and well-accepted drug nanocarriers from laboratories to clinics for their known and predictable biodegradation, avoiding accumulation in the body and thus reducing the toxicity or immunogenicity risks [82]. The extensive investigations on the in vitro and in vivo effects of Apt-2cNP on inhibiting animal colorectal models, thus, show a notable translational potential of Apt-2cNP for clinical benefits.

## Conclusions

We found that a novel BA analogue, 2c, is a potent inhibitor of human colorectal carcinoma. To overcome its off-target cytotoxicity and insolubility and for tumor-specific localization, we encapsulated 2c in PLGA (US-FDA-approved polymer) nanoparticles conjugated with aptamer (Apt-2cNP) that bind EpCAM. Apt-2cNP predominantly induced S-phase arrest, autophagy, and apoptosis in colorectal cancer cells and activated immune cells with a positive anti-tumorigenic impact in the tumor microenvironment. These advantages provide a better in vivo tumor reduction potential for 2c. Overall, this targeted nanotherapy has a high translational potential in treating colorectal cancer. However, further studies are required.

### Supplementary Information

Supplementary Materail 1.

## Data Availability

All data generated in the present study may be requested from the corresponding authors.

## Abbreviations

BA: Betulinic acid
EpCAM: Epithelial cell adhesion molecule
CRC: Colorectal cancer
Apt-2cNP: Aptamer-conjugated 2c-loaded nanoparticle
TME: Tumor microenvironment
ssDNA: Small single-stranded DNA
DMH: Dimethylhydrazine
HPLC: High-performance liquid chromatography
EDC: 1-Ethyl-3-(3-(dimethylamino)propyl)-carbodiimide
NHS: *N*-Hydroxysuccinimide
XPS: X-ray photoelectron spectroscopy
β-CD: β-Cyclodextrin
DLS: Dynamic light scattering
AFM: Atomic force microscopy
FESEM: Field emission scanning electron microscopy
TEM: Transmission electron microscopy
TIase I: Topoisomerase-I
TIase II: Topoisomerase-II
AVO: Acidic vesicular organelles
DC: Dendritic cells
ICD: Immunogenic cell death
mDCs: Myeloid DCs
pDCs: Plasmacytoid DCs
US-FDA: The United States Food and Drug Administration
