# Supplementary material for: EpCAM-targeted betulinic acid analogue nanotherapy improves therapeutic efficacy and induces anti-tumorigenic immune response in colorectal cancer tumor microenvironment
Source: J Biomed Sci. 2024 Aug 20;31:81. doi: 10.1186/s12929-024-01069-8 (PMC11334571; doi:10.1186/s12929-024-01069-8)
Supplement: Supplementary file 1 — Supplementary Materail 1. [file 12929_2024_1069_MOESM1_ESM.pdf]

Supplementary information

EpCAM-targeted betulinic acid analogue nanotherapy improves therapeutic efficacy and induces anti-tumorigenic immune response in colorectal cancer tumor microenvironment

Debasmita Dutta[1,2,3,7,8]*, Ashique Al Hoque[2,3], Brahamacharry Paul[3], Jun Hyoung Park[1], Chinmay Chowdhury[4], Mohiuddin Quadir[2], Soumyabrata Banerjee[3,5], Arghadip Choudhury[4], Soumik Laha[4], Nayim Sepay[6], Priyanka Boro[4], Benny Abraham Kaipparettu[1]*, Biswajit Mukherjee[3]*

[1]Department of Molecular and Human Genetics, Baylor College of Medicine, Houston, Texas, USA

[2]Department of Coatings and Polymeric Materials, North Dakota State University, Fargo, ND, USA.

[3]Department of Pharmaceutical Technology, Jadavpur University, Kolkata, India.

[4]CSIR- Indian Institute of Chemical Biology, Kolkata, India.

[5]Department of Psychology and Neuroscience Program, Central Michigan University, Mount Pleasant, MI 48859, USA.

[6]Department of Chemistry, Jadavpur University, Kolkata, India.

*Corresponding Authors.

Current Address: [7]Dana Farber Cancer Institute, Boston, MA, USA.

[8]Harvard Medical School, Boston, MA, USA.

Supplementary Methods

Cell line and culture maintenance

Adherent type human colorectal carcinoma cell line HT-29 and HCT-116 was procured from the National Center for Cell Science (NCCS, Pune, India) and maintained in a humidified incubator at 37°C in a 5% CO_2 environment using high glucose DMEM medium supplemented with 10% FBS, 100 IU/ml penicillin G, 50 µg/ml streptomycin and 40 µg/ml gentamycin. The cells were cultured in surface-coated cell culture flasks and subcultured in 90% confluency. The viability of the cells was checked using the trypan blue exclusion method whenever necessary.

MTT assay

Briefly, HT-29 and HCT-116 cells were seeded on each well of a 96-well plate at a density of 5-10 $\times 10^4$ cells/well and incubated overnight with supplemented DMEM medium in a humidified incubator at 37°C in 5% CO_2. Then, the media was removed, and the cells were treated with 2c, 2cNP, and Apt-2cNP (treatment volume 200 µl, concentration range 1-50 µmol) for 48 h. A parallel series was run without any drug or formulation and considered a control. After completion of treatment time (48 h), the treatment solution was removed, and the cells were incubated with MTT solution (1 mg/ml, 100 µl in each well) for 4 h. Then the MTT solution was removed, and the cells were dissolved in DMSO (100 µl) to extract the formazan crystals formed in the reaction of MTT with the mitochondrial enzymes of live cells. The absorbance of the DMSO solution was then recorded at 540 nm in an ELISA reader (Bio-Rad, CA, USA). Cell viability % was calculated by comparing it with the control group. IC_{50} value was determined by the intersection method from cell viability (%) vs concentration graph, prepared using Graph Pad Prism software (Graph Pad Prism Inc., San Diego, CA, USA).

In vitro cellular uptake study

HT-29 cells were placed in 60 mm dishes at a density of 1×10^6 per dish and incubated with DMEM media overnight in a humidified incubator. Thereafter, the media was removed, and the cells were treated with Apt-2cNP (which contains Cy5) for 1, 2 and 4 h. Then the

treatment solution was removed, the cells were collected through trypsinization, washed with PBS, re-dispersed in PBS and analyzed in a flow cytometer (BD LSR Fortessa, BD Biosciences) using the channel for Cy5 (Excitation/ Emission 645 nm/ 664 nm). The data was analyzed using FACS Diva software.

For measuring intracellular uptake, around 2×10^4 cells were seeded on a coverslip, placed in a 35 mm tissue culture dish with 1 ml of DMEM media, and incubated overnight at 37°C. The cells were treated with Apt-2cNP for 1, 2, and 4 h. After completion of treatment; the cells were washed with PBS, fixed using 70% ethanol, counter-stained with DAPI (for staining nucleus), and mounted on a slide using Prolong Diamond Antifade mountant. The cells were then observed under a confocal laser microscope (Olympus FluoView FV10i, Olympus) using the filters for Cy5 (Excitation/ Emission 645 nm/ 664 nm) and DAPI (Excitation /Emission 359 nm/461 nm). Images were captured separately for each channel and merged using the Olympus FluoView software to get dual-color images.

Supplementary Figures:

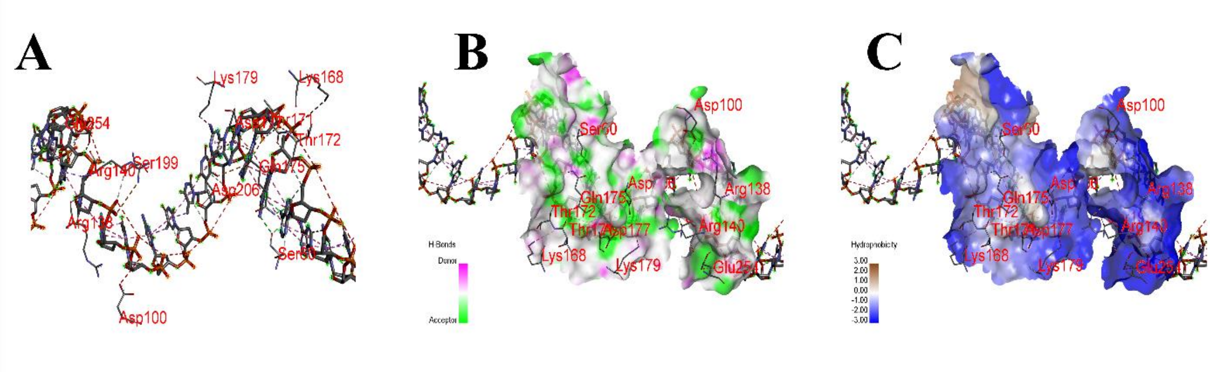

Supplementary Figure S1: Aptamer-EpCAM (PDB: 4MZV) interactions through molecular docking analysis (A) binding of various amino acid residues with the DNA aptamer, **(B)** hydrogen bonding interactions between aptamer and EpCAM, and **(C)** hydrophobic interactions between aptamer and EpCAM

2c

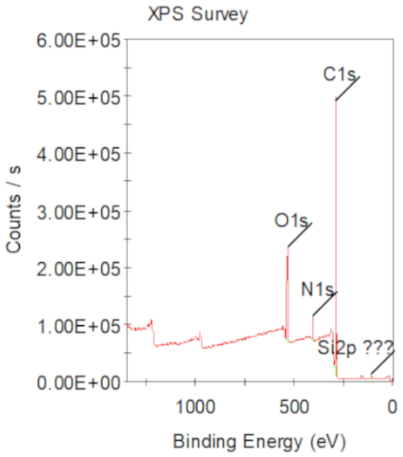

	2c		
	C1s	O1s	N1s
Peak BE (eV)	284.89	532.84	400.77
Height (CPS)	4444028.94	160940.72	41877.72
Area (CPS.eV)	1448851.68	551253.14	146108.35
Atomic %	80.75	12.72	5.25

2cNP

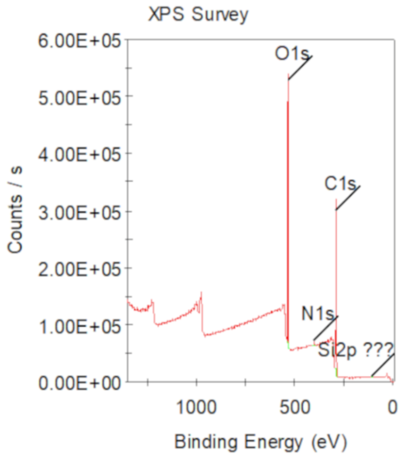

	2cNP		
	C1s	O1s	N1s
Peak BE (eV)	285.85	533.19	401.18
Height (CPS)	287459.51	449538.97	9966.02
Area (CPS.eV)	1341517.16	1538750.69	45046.93
Atomic %	66.52	31.58	1.44

Apt-2cNP

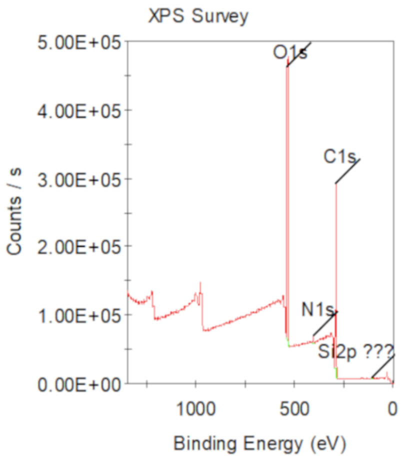

	Apt-2cNP		
	C1s	O1s	N1s
Peak BE (eV)	286.01	533.5	401.26
Height (CPS)	280061.75	400112.29	11955.56
Area (CPS.eV)	1299855.65	1436960.88	54124.7
Atomic %	67	30.66	1.8

Supplementary Figure S2: XPS data analysis of 2c, 2cNP and Apt-2cNP.

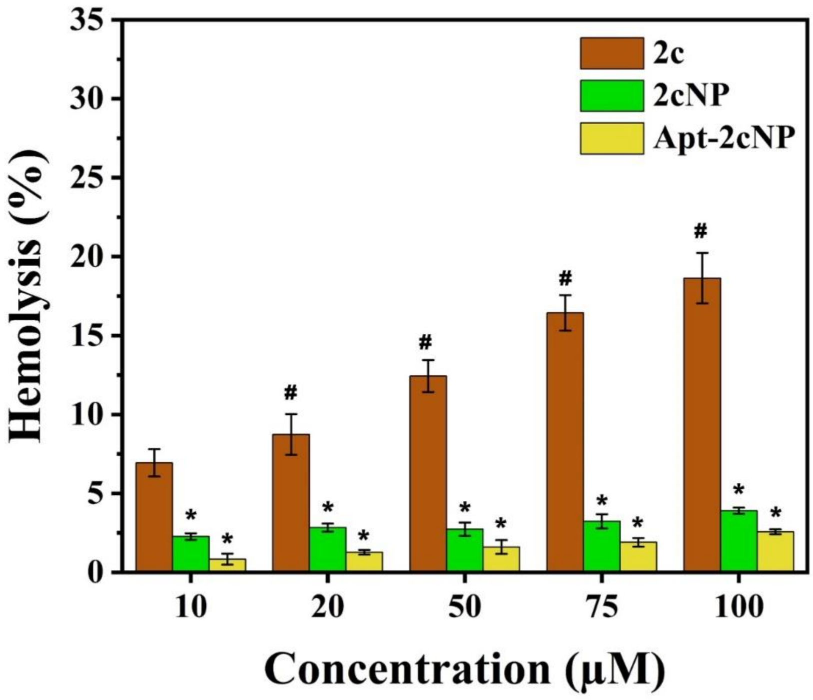

Supplementary Figure S3: Evaluation of systemic safety profile by hemolytic analysis. The hemolytic activities of 2c, 2cNP, and Apt-2cNP were assessed by subjecting each sample to incubation with red blood cells, followed by quantification of the resultant hemoglobin released. Subsequently, the percentage of hemolysis was plotted against the concentration of the samples, enabling the evaluation of their hemolytic potential. Data shows mean ± SD (n = 3) , where * $p<0.05$ when compared against individual **2c** control group, and # $p<0.05$ when **2c** control groups were compared with **2c** control group treated with 10 µM **2c**.

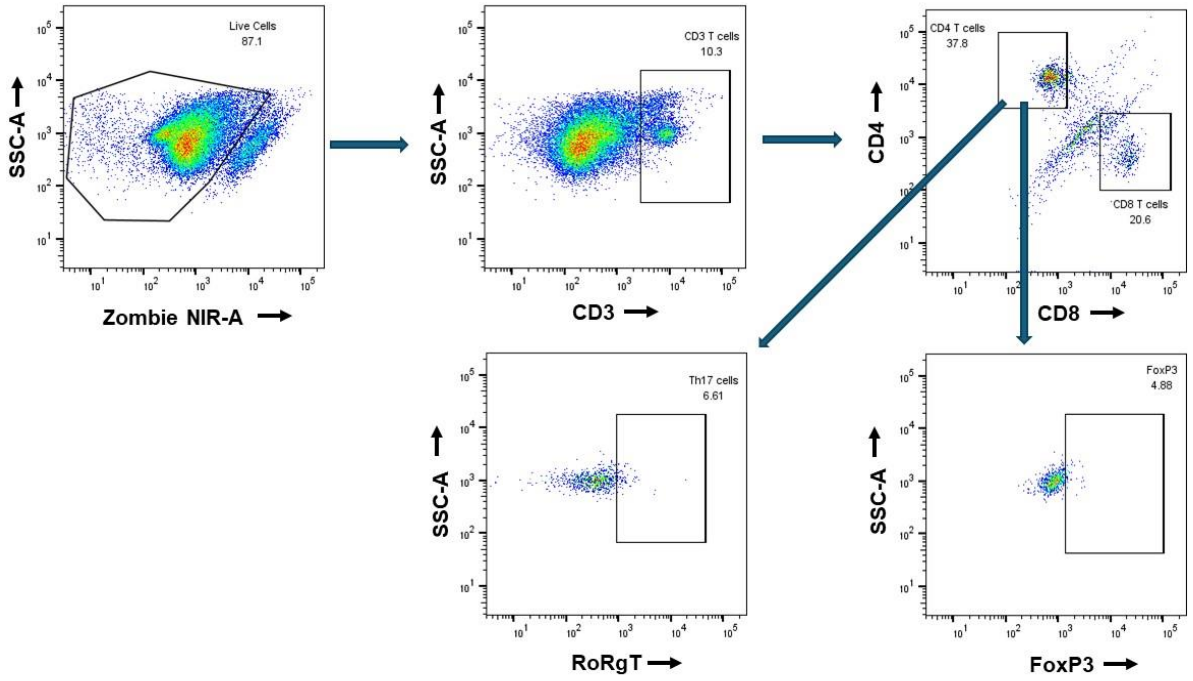

Supplementary Figure S4: Flow gating strategy. Representative gating used to study T cells population for CRC murine model. Live cells population was first gated out using the live-dead cells staining with zombie-NIR followed by CD3+ population was analyzed for CD4+ cells and CD8+ T cells. The CD3+CD4+ T cell population was further subjected to analyzing the expression of Th17 and FOXP3 cells.

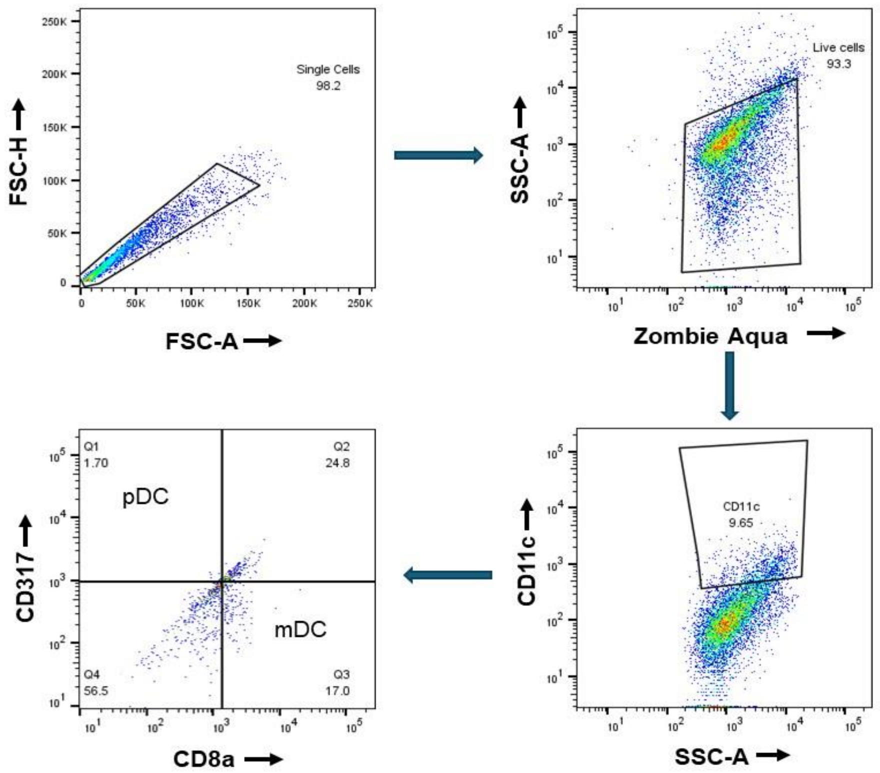

Supplementary Figure S5: Representative flow gating strategy used for dendritic cells in tumor microenvironment of CRC sample. The live single cells were first gated out for live-dead staining, followed by mDC, and pDC population was analyzed on CD11c+ dendritic cells.

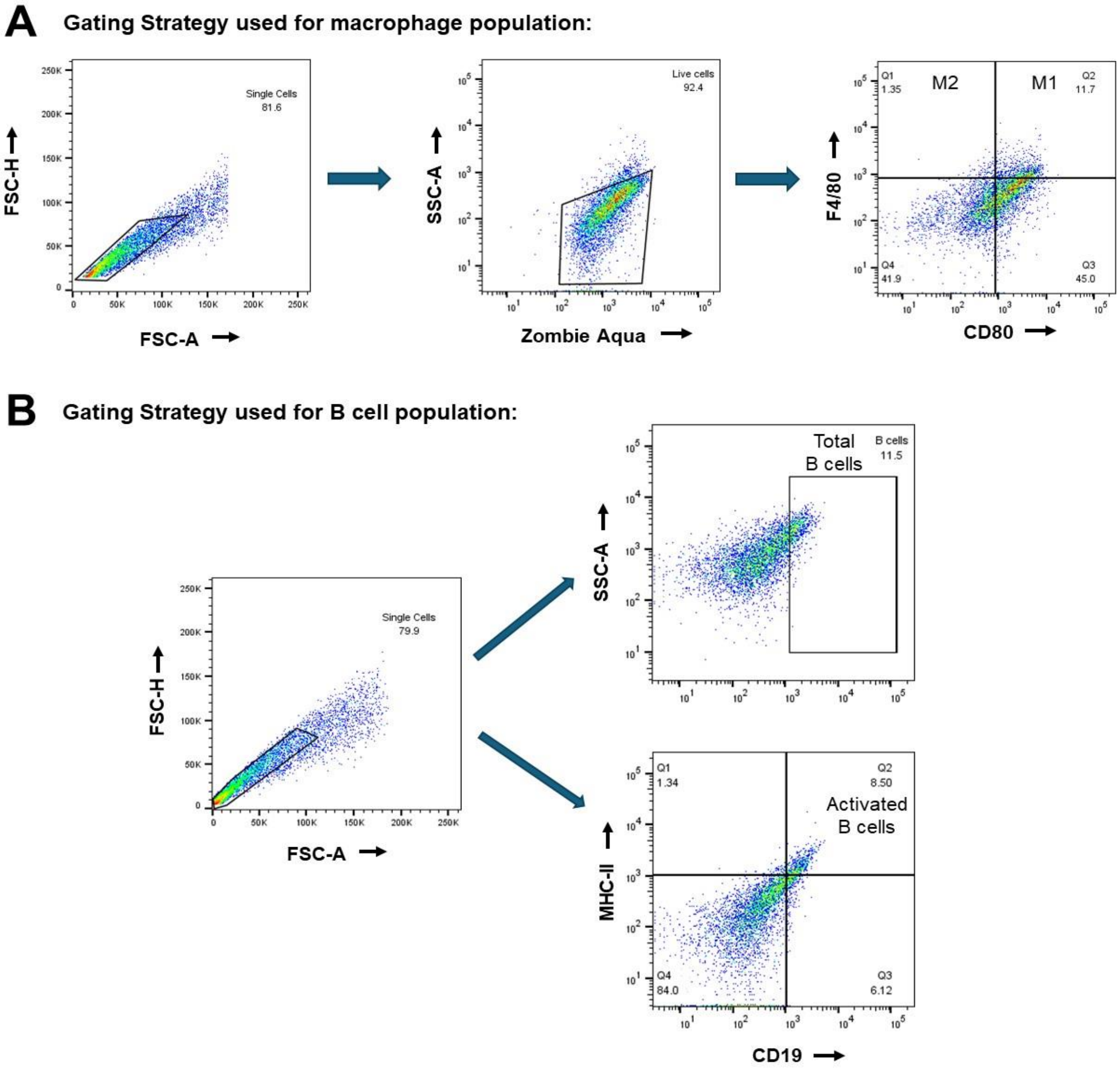

Supplementary Figure S6: Representative flow cytometry gating strategy used for macrophages and B cells in tumor microenvironment of CRC sample. (A) The live single cells were first gated out for live-dead staining, followed by M1 and M2 populations of macrophages. **(B)** The live single cells were first gated out, and total B cells and activated B cell populations were analyzed on the live cell population.

Supplementary Tables:

Supplementary Table T1: Animal groups and specifications

**Animal Group**	**Number of animals in each group**	**Specification**
Group I - Normal control	Five mice	Received normal food and water *ad libitum*
Group II - Carcinogen control	Five rats and twenty five mice	Received carcinogen (DMH) with normal food and water *ad libitum*
Group III- Carcinogen mice treated with **2c**	Five rats and twenty five mice	Received **2c** after completion of carcinogen treatment, along with normal food and water *ad libitum*
Group IV- Carcinogen treated with **2cNP**	Five rats and twenty five mice	Received **2c-NP** after completion of carcinogen treatment, along with normal food and water *ad libitum*
Group V – Carcinogen treated with **Apt-2cNP**	Five rats and twenty five mice	Received **Apt-2cNP** after completion of carcinogen treatment along with normal food and water *ad libitum*

Supplementary Table T2: Sequences of primers used in PCR analysis

**Gene**	F/R	**Primer sequence**
Caspase-3	F	5′-TGA CTG GAA AGC CGA AAC TC-3′
	R	5′-AGC CTC CAC CGG TAT CTT CT-3′
Bax	F	5′-CCC GAG AGG TCT TTT TCC-3′
	R	5′-GCC TTG AGC ACC AGT TTG-3′
Bcl-2	F	5′-CCT GTG GAT GAC TGA GTA CCT G-3′
	R	5′-AGC CAG GAG AAA TCA AAC AGA GG -3′

Bcl-XL	F	5′-AAC ATC CCA GCT TCA CAT AAC CCC-3′
	R	5′-GCG ACC CCA GTT TAC TCC ATC C-3′
Beclin	F	5′-CAG CCT CTG AAA CTG GAC ACG A-3′
	R	5′-CTC TCC TGA GTT AGC CTC TTC C-3′
Atg5	F	5′-CTT GCA TCA AGT TCA GCT CTT CC-3′
	R	5′-AAG TGA GCC TCA ACC GCA TCC T-3′
LC3B	F	5′-GTC CTG GAC AAG ACC AAG TTC C-3′
	R	5′-CCA TTC ACC AGG AGG AAG AAG G-3′
p62	F	5′-ACA CCT GCT TCT GGA GGA ACA G-3′
	R	5′-TTG GAG GTG CTG CCA CTT GAG A-3′
p53	F	5′-CTG GTT AGT CCT GAG ACA GAG G-3′
	R	5′-AGA TGC AGC CAA ACA CAG GCA C-3′
APC	F	5′-AGC CAT GCC AAC AAA GTC ATC ACG-3′
	R	5′-TTC CTT GCC ACA GGT GGA GGT AAT-3′
NF-κB	F	5′-GAA ATT CCT GAT CCA GAC AAA AAC-3′
	R	5′-ATC ACT TCA ATG GCC TCT GTG TAG-3′
TOP2	F	5′-GCT GAG ATG TCT GCT TAC CAC C-3′
	R	5′-GCA TCT TTG CCA CCA TGA AGC C-3′
GAPDH	F	5′-CAT CAC TGC CAC CCA GAA GAC TG-3′
	R	5′-ATG CCA GTG AGC TTC CCG TTC AG-3′

F = Forward sequence, R = reverse sequence.

Supplementary Table T3: Drug loading (%), encapsulation efficiency (%), particle size (nm), and zeta potential (mV) of 2cNP and Apt-2cNP

**Parameter**	**2cNP**	**Apt-2cNP**
Drug loading (%)	8.0±0.5 %	7.9±0.2 %
Encapsulation efficiency (%)	86 %	87 %
Particle size (nm)	180 ± 15 nm	188 ± 14 nm
Zeta potential (mV)	-7.94 ± 1 mV	-11.5 ± 0.5 mV

Supplementary Table T4: Aptamer-EPCAM binding using molecular docking analysis

Name of Protein	PDB code	Aptamer sequence	Docking Score	Interacting amino acid residues	Type of interactions
Epithelial cell adhesion molecule (EpCAM)	4MZV	5′-ATCCAGAGTG ACGCAGCATGCG GCACACACTTCT ATCTTTGCGGAA CTCCTGCGGCTC TGGACACGGTG GCTTAGT-3’	-254.23	Ser60, Ser199 Asp100, Asp177, Asp206, Gln175, Arg138, Arg140, Glu254 Lys168, Lys179 Thr171, Thr172	Electrostatic interactions, Hydrogen bonding

Supplementary Table T5: Drug release profile of 2c from Apt-2cNP as explained through different kinetic models. Regression coefficient values (R^2) and representative equations as obtained using different kinetic models

In vitro drug release kinetics models	Apt-2cNP					
	In PBS, pH 7.4		In PBS, pH 7.4 + 0.5% β-cyclodextrin		In Acetate Buffer, pH 5.5	
	R^2 Value	Representing Equations	R^2 Value	Representing Equations	R^2 Value	Representing Equations
Zero Order	0.846	y = 0.059x + 16.22	0.819	y = 0.059x + 21.58	0.655	y = 0.089x + 28.76
First Order	0.960	y = -0.000x + 1.929	0.940	y = -0.000x + 1.883	0.939	y = -0.001x + 1.850

Higuchi	0.963	y = 2.352x + 6.072	0.951	y = 2.374x + 11.10	0.838	y = 3.060x + 16.91
Hixon Crowell	0.931	y = 0.001x + 0.260	0.893	y = 0.001x + 0.406	0.857	y = 0.003x + 0.527
Korsmeyer Peppas	0.932	y = 0.514x + 0.441	0.941	y = 0.402x + 0.782	0.895	y = 0.420x + 0.892

Supplementary Table T6: Pharmacokinetic parameters after treating mice with 2c/ 2cNP/ Apt-2cNP

**Parameters**	**2c**	**2cNP**	**Apt-2cNP**
**Cmax (ng/ml)**	23.5 ± 0.55	18.4 ± 0.58	20.4 ± 0.47
**Tmax (h)**	1 min	8.00 ± 0.2	8.00 ± 0.2
**AUC last (ng.h/ml)**	376 ± 42	819 ± 43	971 ± 47
**$t_{1/2}$ (h)**	6.0 ± 0.5	34.0 ± 1.5	36.0 ± 1.0
**MRT (h)**	22.3 ± 1.72	35.4 ± 1.89	37.6 ± 1.48
**AUMC (ng.h^2/ml)**	8381 ± 1644	29036 ± 1810	36559 ± 2104
**AUC $_{0-\infty}$ (ng. h/ml)**	380 ± 44	1020 ± 57	1273 ± 68
**Clearance (ml/h)**	333 ± 30	152 ± 20	128 ± 15
**Vd (L)**	2.88 ± 0.15	7.46 ± 0.35	6.67 ± 0.26

Supplementary Table T7: Biodistribution of ^{99m}Tc-Apt-2cNP and ^{99m}Tc-2cNP in CRC rats at different time intervals

Organ/Tissue	**%ID of ^{99m}Tc-Apt-2cNP in CRC rats (SD)**			**%ID of ^{99m}Tc-2cNP in CRC rats (SD)**		
	**1 h**	**2 h**	**5 h**	**1 h**	**2 h**	**5 h**
Blood#	2.883 ± 0.434	2.122 ± 0.346	1.933 ± 0.124	2.166 ± 0.115	1.708 ± 0.099	0.854 ± 0.045
Heart	1.332 ± 0.116	1.541 ± 0.121	1.722 ± 0.144	1.516 ± 0.098	1.602 ± 0.096	1.711 ± 0.078
Liver	35.36 ± 3.325 *	30.48 ± 2.682 *	27.44 ± 3.585	42.21 ± 3.185 *	36.55 ± 3.101 *	27.82 ± 2.885
Lungs	1.624 ±	1.924 ±	1.845 ±	1.112 ±	1.668 ±	1.905 ±

	0.321	0.227	0.356	0.115	0.118	0.125
Stomach	1.456 ± 0.188	1.563 ± 0.132	1.484 ± 0.212	1.225 ± 0.117	1.286 ± 0.121	1.327 ± 0.122
Spleen	2.213 ± 0.422	3.242 ± 0.378	2.766 ± 0.588	1.686 ± 0.128	1.884 ± 0.124	1.938 ± 0.138
Intestine	6.724 ± 0.415	8.346 ± 0.274	11.42 ± 0.329 *	3.012 ± 0.330	5.511 ± 0.748	6.007 ± 0.556 *
Kidney	7.248 ± 1.559	12.34 ± 2.455 *	16.55 ± 2.728 *	6.882 ± 1.341	7.140 ± 0.883 *	5.711 ± 0.689 *
Urine	13.46 ± 2.825	19.45 ± 3.662	24.36 ± 4.611 *	17.02 ± 2.102	22.14 ± 2.518	29.16 ± 4.588 *
Muscle#	0.061 ± 0.005	0.071 ± 0.011	0.074 ± 0.009	0.048 ± 0.009	0.060 ± 0.007	0.055 ± 0.011

The values were calculated as a percentage of injected dose present in each organ (%ID), and in the case of blood and muscle (marked as #) these values were calculated as %ID per gram of tissue (%ID/g). Asterisk marks (*) represent statistically significant values when both **^{99m}Tc-Apt-2cNP** and **^{99m}Tc-2cNP** were compared for different time points by Two-way ANOVA analysis and tested by Bonferoni’s post-test ($p<0.05$).
